# Transforming Growth Factor‐β and Axl Induce CXCL5 and Neutrophil Recruitment in Hepatocellular Carcinoma

**DOI:** 10.1002/hep.30166

**Published:** 2018-12-20

**Authors:** Christine Haider, Julia Hnat, Roland Wagner, Heidemarie Huber, Gerald Timelthaler, Markus Grubinger, Cédric Coulouarn, Wolfgang Schreiner, Karin Schlangen, Wolfgang Sieghart, Markus Peck‐Radosavljevic, Wolfgang Mikulits

**Affiliations:** ^1^ Department of Medicine I, Institute of Cancer Research, Comprehensive Cancer Center Vienna Medical University of Vienna Vienna Austria; ^2^ INSERM, University of Rennes, INRA, Institute NUMECAN (Nutrition Metabolisms and Cancer) UMR_A 1341, UMR_S 1241 Rennes France; ^3^ Division of Biosimulation and Bioinformatics, Center for Medical Statistics, Informatics and Intelligent Systems (CeMSIIS) Medical University of Vienna Vienna Austria; ^4^ Department of Internal Medicine III, Division of Gastroenterology and Hepatology Medical University of Vienna Vienna Austria

## Abstract

Transforming growth factor (TGF)‐β suppresses early hepatocellular carcinoma (HCC) development but triggers pro‐oncogenic abilities at later stages. Recent data suggest that the receptor tyrosine kinase Axl causes a TGF‐β switch toward dedifferentiation and invasion of HCC cells. Here, we analyzed two human cellular HCC models with opposing phenotypes in response to TGF‐β. Both HCC models showed reduced proliferation and clonogenic growth behavior following TGF‐β stimulation, although they exhibited differences in chemosensitivity and migratory abilities, suggesting that HCC cells evade traits of anti‐oncogenic TGF‐β. Transcriptome profiling revealed differential regulation of the chemokine CXCL5, which positively correlated with TGF‐β expression in HCC patients. The expression and secretion of CXCL5 was dependent on Axl expression, suggesting that CXCL5 is a TGF‐β target gene collaborating with Axl signaling. Loss of either TGF‐β or Axl signaling abrogated CXCL5‐dependent attraction of neutrophils. In mice, tumor formation of transplanted HCC cells relied on CXCL5 expression. In HCC patients, high levels of Axl and CXCL5 correlated with advanced tumor stages, recruitment of neutrophils into HCC tissue, and reduced survival. *Conclusion:* The synergy of TGF‐β and Axl induces CXCL5 secretion, causing the infiltration of neutrophils into HCC tissue. Intervention with TGF‐β/Axl/CXCL5 signaling may be an effective therapeutic strategy to combat HCC progression in TGF‐β‐positive patients.

**Abbreviations**
EGF: epidermal growth factor
HCC: hepatocellular carcinoma
MAPK: mitogen‐activated protein kinase
NET: neutrophil extracellular trap
PI3K: phosphoinositide 3 kinase
RTK: receptor tyrosine kinase
siRNA: small interfering RNA
Smad3L: Smad3 linker
TGF: transforming growth factor

Globally, liver cancer is the fifth most frequently diagnosed cancer and the second most frequent cause of cancer‐related deaths.^1^ Hepatocellular carcinoma (HCC) accounts for 70% to 85% of the total liver cancer burden worldwide. The main reasons for the high mortality rate of HCC patients are diagnosis at an advanced stage and intrahepatic metastasis.^2^ Approximately 80% of HCC develop on a cirrhotic background caused by chronic infection with hepatitis B or C virus, metabolic disorders, nonalcoholic steatohepatitis, or alcohol intoxication.^3^

Chronic inflammation caused by these conditions leads to cell death, compensatory liver regeneration, and activation of nonparenchymal cells that promotes liver fibrosis and tumorigenesis.^4^ Alterations in the immune response involve the infiltration of adaptive and innate immune cells, producing a pathological milieu composed of multiple extracellular matrix proteins, growth factors, and chemokines that can form a protumorigenic stroma.^3^, ^5^ It has been proposed that neutrophil infiltration is prognostic in several human cancers,^6^ including HCC.^7^ Neutrophils influence tumor progression through the paracrine release of cytokines and chemokines with protumorigenic or antitumorigenic functions, depending on the tumor microenvironment.^8^

Transforming growth factor (TGF)‐β is a key profibrogenic cytokine that is predominantly produced by activated mesenchymal cells following chronic liver damage.^9^, ^10^ TGF‐β signals through a formation of a heterotetrameric complex of type I and type II serine/threonine kinase receptors following ligand binding, which then cause canonical signaling by C‐terminal phosphorylation of Smad2 and Smad3 as well as complex formation with Smad4.^11^ The activated Smad complexes translocate into the nucleus, where they regulate the transcription of multiple target genes in cooperation with co‐activators and corepressors. A multitude of signaling pathways can be activated by TGF‐β in a Smad‐independent manner through direct phosphorylation of downstream effectors, including the Ras/mitogen‐activated protein kinase (MAPK), c‐Jun N‐terminal kinase, p38 MAPK, Cdc42, Par6, as well as the phosphoinositide 3 kinase (PI3K)/Akt pathway.^12^

Cellular responses to TGF‐β signaling result from the dynamic combination of canonical and noncanonical signaling cascades and from the crosstalk with other signaling pathways. In HCC, TGF‐β acts anti‐oncogenically in normal hepatocytes and early carcinomas, yet cytostatic and cytotoxic effects are frequently lost after progression, leading to invasion and metastasis.^13^, ^14^ Tumor‐promoting TGF‐β signaling has been shown to depend on receptor tyrosine kinase (RTK) signaling such as epidermal growth factor (EGF)/EGFR and hepatocyte growth factor/Met as well as integrins, which allows chemoresistance and escape from TGF‐β/Smad‐mediated apoptosis.^15^, ^16^, ^17^ Our recent study showed that signaling from the RTK Axl is central for TGF‐β‐mediated HCC progression. Axl signaling causes the aberrant phosphorylation of the Smad3 linker (Smad3L) region and induction of prometastatic target genes as well as increased secretion of TGF‐β1,^18^ which has been shown to have a large impact on the immune cells in the tumor microenvironment.^19^ Despite tremendous efforts and large progress in cancer research, the molecular mechanisms underlying this “TGF‐β switch” in HCC are still poorly understood.

In this study, we mimicked the tumor‐promoting role of TGF‐β by establishing cellular HCC models that were long‐term exposed to TGF‐β, allowing us to identify molecular mechanisms that may cause the TGF‐β switch. From these genes, we focused on CXCL5, which belongs to the CXC chemokine family that is also known as epithelial‐derived neutrophil‐activating peptide 78, binding primarily to the chemokine receptor CXCR2.^20^ Most notably, we found that CXCL5 is regulated by the collaboration of TGF‐β and Axl signaling to facilitate neutrophil infiltration in HCC patients.

## Materials and Methods

To analyze CXCL5, elastase, Axl, TGF‐β1, and phospho‐Smad3L expression in primary HCC, tissue arrays were used that contained paraffin‐embedded specimens of tumors and adjacent normal tissue collected from 133 HCC patients. All patients have undergone orthotopic liver transplantation for HCC at the Department of Transplantation Surgery, Medical University of Vienna, between 1982 and 2002, as described.^18^ All histological specimens were reviewed for histological type and graded by two individual board‐certified pathologists. Detailed information on experimental procedures can be found in the Supporting Information.

## Results

### Autocrine TGF‐β Regulation and Cell Migration of Mesenchymal‐like HCC Cells

**Figure 1:**
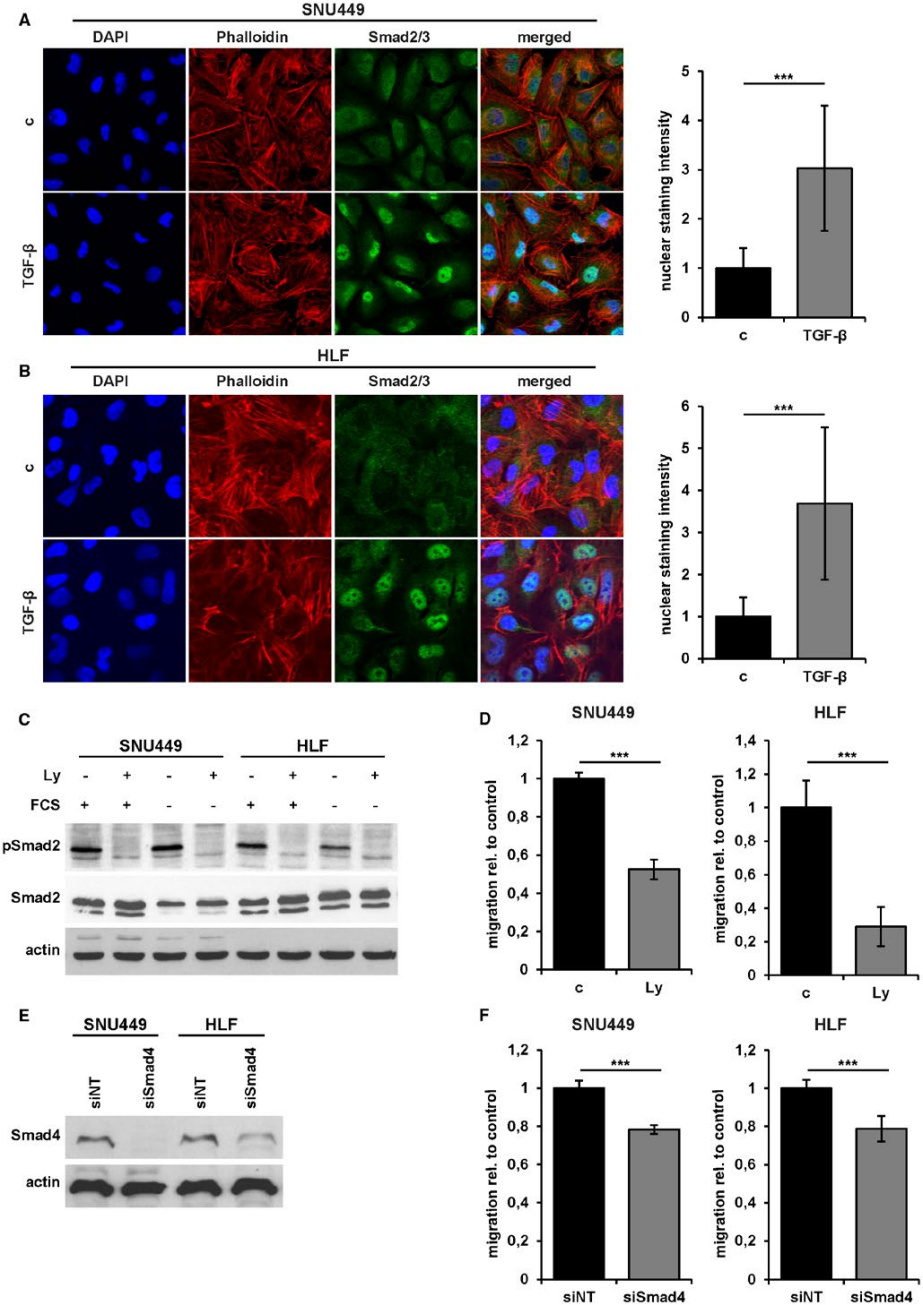
Role of TGF‐β in mesenchymal‐like HCC cells. (A) Left panel: Confocal immunofluorescence analysis of Smad2/3 in SNU449 cells treated with 2.5 ng/mL TGF‐β1 for 15 minutes. Actin stress fibers are indicated by phalloidin staining (red). Nuclei were counterstained with DAPI (blue). Right panel: Quantification of nuclear Smad2/3 signal intensity using ImageJ. (B) Immunofluorescence analysis of HLF cells as described in (A). (C) Western blot analysis of pSmad2 and total Smad2/3, with and without FCS, and interference with 10 µM Ly for 24 hours. Actin was used as loading control. (D) Migrated areas of HLF and SNU449 cells and those treated with 10 µM Ly in wound healing assays. (E) Levels of Smad4 after treatment with siNT or siSmad4. (F) Migrated areas of HLF and SNU449 cells treated with siNT or siSmad4. Data are expressed as mean ± SD. Error bars depict SD from at least three individual experiments. ***P < 0.001. Abbreviations: c, untreated control; FCS, fetal calf serum.

Our recent study showed that dedifferentiated, mesenchymal‐like HCC cell lines secrete more TGF‐β than epithelial HCC cells and exhibit higher migratory abilities.^18^ Two of these mesenchymal‐like cell lines, SNU449 and HLF (hepatic leukemia factor), were further investigated and exploited as cellular models. To investigate the role of the TGF‐β signaling, we first demonstrated that these cell lines have an active TGF‐β signaling by Smad2/3 translocation into the cell nucleus after short‐term TGF‐β treatment (Fig. 1A,B; left panels). SNU449 and HLF cells treated with TGF‐β showed a 3‐fold and 3.7‐fold increase in nuclear staining intensity, respectively (Fig. 1A,B; right panels). Inhibition of TGF‐β through LY2109761 (Ly)^21^ abrogated autocrine stimulation (Fig. 1C), whereas TGF‐β/Smad signaling remained unaffected after FCS stimulation. To examine the role of TGF‐β on cell motility, cells were treated with Ly and analyzed by wound healing assays (Fig. 1D). SNU449 and HLF cells showed a 50% and 70% decrease in migration after TGF‐β inhibition, respectively, suggesting that both cell types were dependent on TGF‐β in their migratory behavior. To confirm these data, we performed a knockdown of Smad4 (Fig. 1E). In accordance, both cell lines showed a 22% reduction in migration (Fig. 1F). These data suggest that autocrine TGF‐β/Smad signaling is crucially involved in the migration of SNU449 and HLF cells.

### Duration‐Dependent and Concentration‐Dependent Migratory Response to TGF‐β Treatment in Mesenchymal‐like HCC Cells

**Figure 2:**
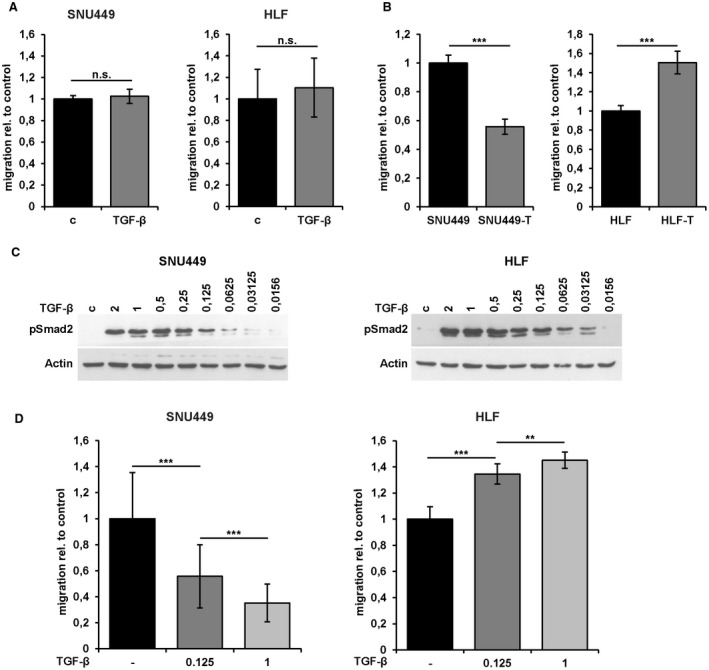
Duration‐dependent and concentration‐dependent migratory response of mesenchymal‐like HCC cells to TGF‐β treatment. (A) Migrated areas of HLF and SNU449 cells and those treated with 2.5 ng/mL TGF‐β1 for 24 hours in wound healing assays. (B) Migrated areas of SNU449 and HLF cells and those long‐term treated with 1 ng/mL TGF‐β1 (> 10 days, termed SNU449‐T and HLF‐T) in wound healing assays. (C) Western blot analysis of pSmad2 after long‐term treatment (> 10 days) of cells with different concentrations of TGF‐β1 (ng/mL). (D) Migrated areas of SNU449 cells (left panel) and HLF cells (right panel) after long‐term treatment (> 10 days) with 0.125 ng/mL and 1 ng/mL TGF‐β1. Data are expressed as mean ± SD. Error bars depict SD from at least three individual experiments. **P < 0.01, ***P < 0.001. Abbreviations: c, control; n.s., not significant.

Although short‐term TGF‐β treatment (24 hours) of SNU449 and HLF cells failed to enhance migratory abilities (Fig. 2A), long‐term TGF‐β treatment (> 10 days) revealed strong differences between the mesenchymal‐like HCC cell lines termed SNU449‐T and HLF‐T cells (Fig. 2B; Supporting Fig. S1). Whereas HLF‐T cells showed a 50% increase in migration, SNU449‐T cells displayed a 50% decrease in migration (Fig. 2B). Cells were further treated with a serial dilution of TGF‐β to assess the minimum concentration required to trigger this phenotype (Fig. 2C). Both cell lines showed strongly phosphorylated Smad2 at a concentration of 0.125 ng/mL TGF‐β. Hence, cells were treated long‐term with 1 ng/mL and 0.125 ng/mL TGF‐β and analyzed for migratory abilities. SNU449 cells administrated with 0.125 ng/mL and 1 ng/mL showed a concentration‐dependent reduction of the migrated area (Fig. 2D, left panel, i.e., 50% and 65%, respectively). Accordingly, HLF cells exhibited a concentration‐dependent increase in migration after treatment with 0.125 ng/mL and 1 ng/mL TGF‐β, i.e., 34% and 45% (Fig. 2D, right panel), respectively. These data indicate that the duration and concentration of TGF‐β are critical parameters for the migratory behavior of the cells. Furthermore, we conclude that long‐term TGF‐β exposure of HCC cells leads to a different use of the TGF‐β pathway through collaboration with other signaling mechanisms.

### Different Chemosensitivity of Long‐Term TGF‐β‐Treated Mesenchymal‐like Cells

**Figure 3:**
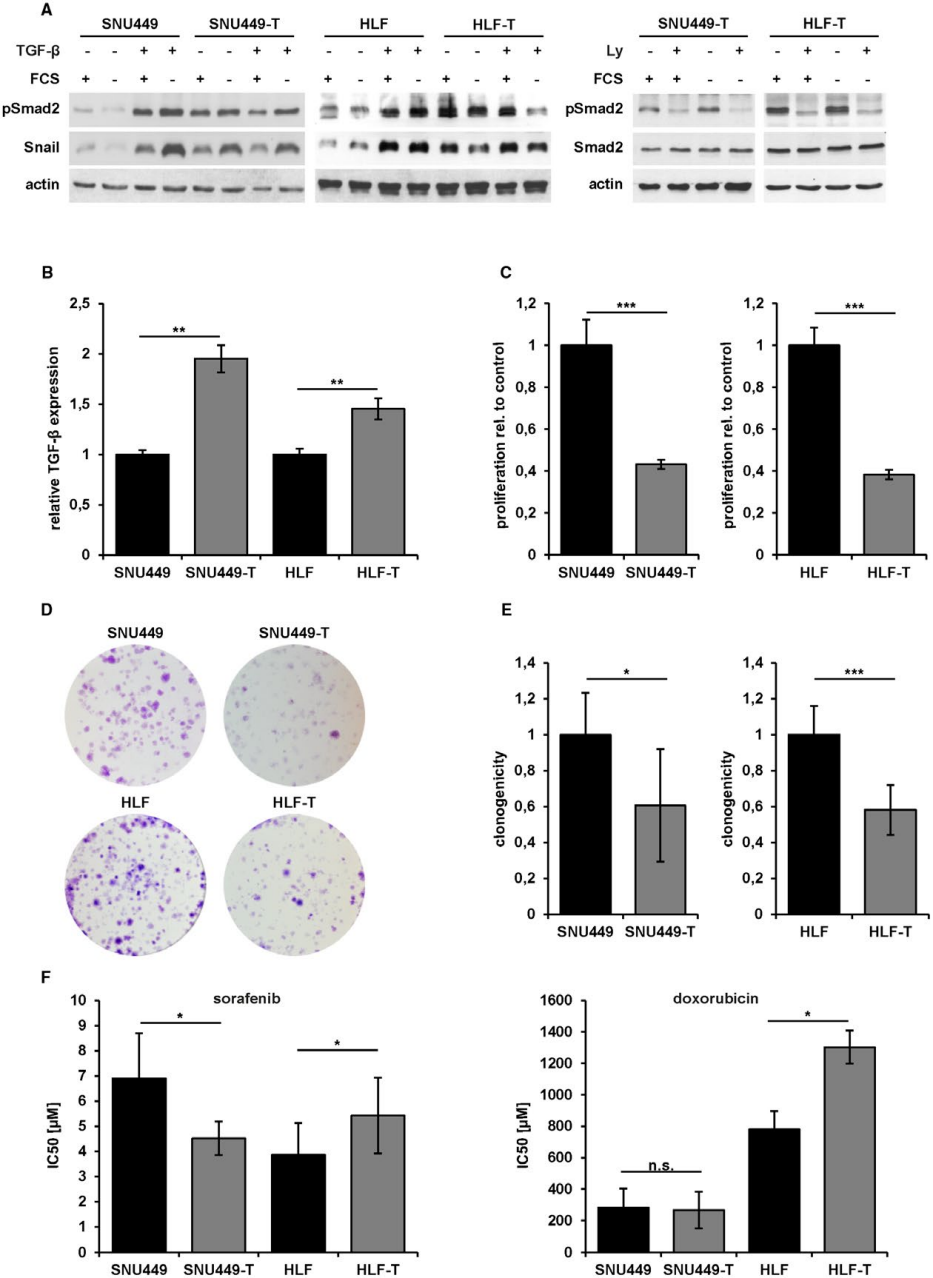
Effects of long‐term TGF‐β exposure on mesenchymal‐like HCC cells. (A) Western blot analysis of pSmad2 and Snail after serum starvation and stimulation with 2.5 ng/mL TGF‐β1 (left panel), and after serum starvation and treatment with 10 µM Ly for 24 hours (right panel). Actin was used as loading control. (B) Analysis of TGF‐β mRNA expression by qPCR. (C) Proliferation kinetics of SNU449/SNU449‐T and HLF/HLF‐T cells over 72 hours. (D) Clonogenic survival assay of SNU449/SNU449‐T (upper panel) and HLF/HLF‐T cells (lower panel) after long‐term treatment with TGF‐β1. Representative images are shown. (E) Quantification of clonogenic survival assay shown in (D). (F) IC_50_ values [µM] of sorafenib (left panel) and doxorubicin (right panel) in SNU449/SNU449‐T and HLF/HLF‐T cells. Data are expressed as mean ± SD. Error bars depict SD from at least three individual experiments. *P < 0.05, **P < 0.01, ***P < 0.001. Abbreviations: n.s., not significant; qPCR, quantitative reverse‐transcriptase polymerase chain reaction.

We further investigated the phosphorylation of Smad2 in SNU449 and HLF cells after long‐term treatment with TGF‐β (>10 days). Notably, the TGF‐β signaling activity was prolonged in both long‐term‐treated SNU449‐T and HLF‐T cells along with the expression of the TGF‐β target gene Snail (Fig. 3A, left panel). Treatment with Ly diminished phosphorylated Smad2 levels in both serum‐starved SNU449‐T and HLF‐T cells, indicating autocrine TGF‐β regulation (Fig. 3A, right panel). In addition, the expression of TGF‐β was higher in SNU449‐T (95%) and HLF‐T (45%) cells in comparison to their parental cells (Fig. 3B). These data show that both cell lines displayed no different modulation in Smad2 phosphorylation or TGF‐β1 expression, suggesting an adaptive response in using TGF‐β signaling. Interestingly, SNU449‐T cells showed a 57% reduction of proliferation compared with SNU449 cells, and HLF‐T showed a 62% decrease compared with HLF cells (Fig. 3C), which indicates reduced proliferation following TGF‐β stimulation. To further evaluate the role of long‐term TGF‐β treatment, the clonogenic growth behavior was analyzed. Both cell lines showed reduced clonogenic abilities with a decline of 40% (Fig. 3D,E). However, cell viability assays after treatment with sorafenib or doxorubicin revealed different responses in these cellular models. SNU449‐T cells showed decreased viability towards sorafenib with IC_50_ values of 4.5 µM and 7 µM in comparison to SNU449 cells, respectively (Fig. 3F, left panel). In contrast, HLF‐T cells showed higher viability in comparison to control cells with IC_50_ values of 5.4 µM and 3.9 µM, respectively (Fig. 3F, left panel). Doxorubicin showed a similar pattern, yet no significant changes of IC_50_ values between SNU449 and SNU449‐T cells were observed (Fig. 3F, right panel). Doxorubicin‐treated HLF‐T cells also showed increased cell viability with an IC_50_ value of 1.300 µM versus 781 µM for HLF cells. These data suggest that both HCC cell types do not evade TGF‐β‐induced tumor‐suppressive traits such as the cytostatic program. However, the same treatment conditions of hepatoma cells that displayed no modulation of TGF‐β/Smad activation allowed HLF‐T cells to escape from drug‐induced cytotoxicity and to induce cell migration, suggesting different use of active TGF‐β signaling rather than its inactivation.

### Transcriptome Profiling of Long‐Term TGF‐β‐Treated HCC Models Reveals HCC Target Genes

**Figure 4:**
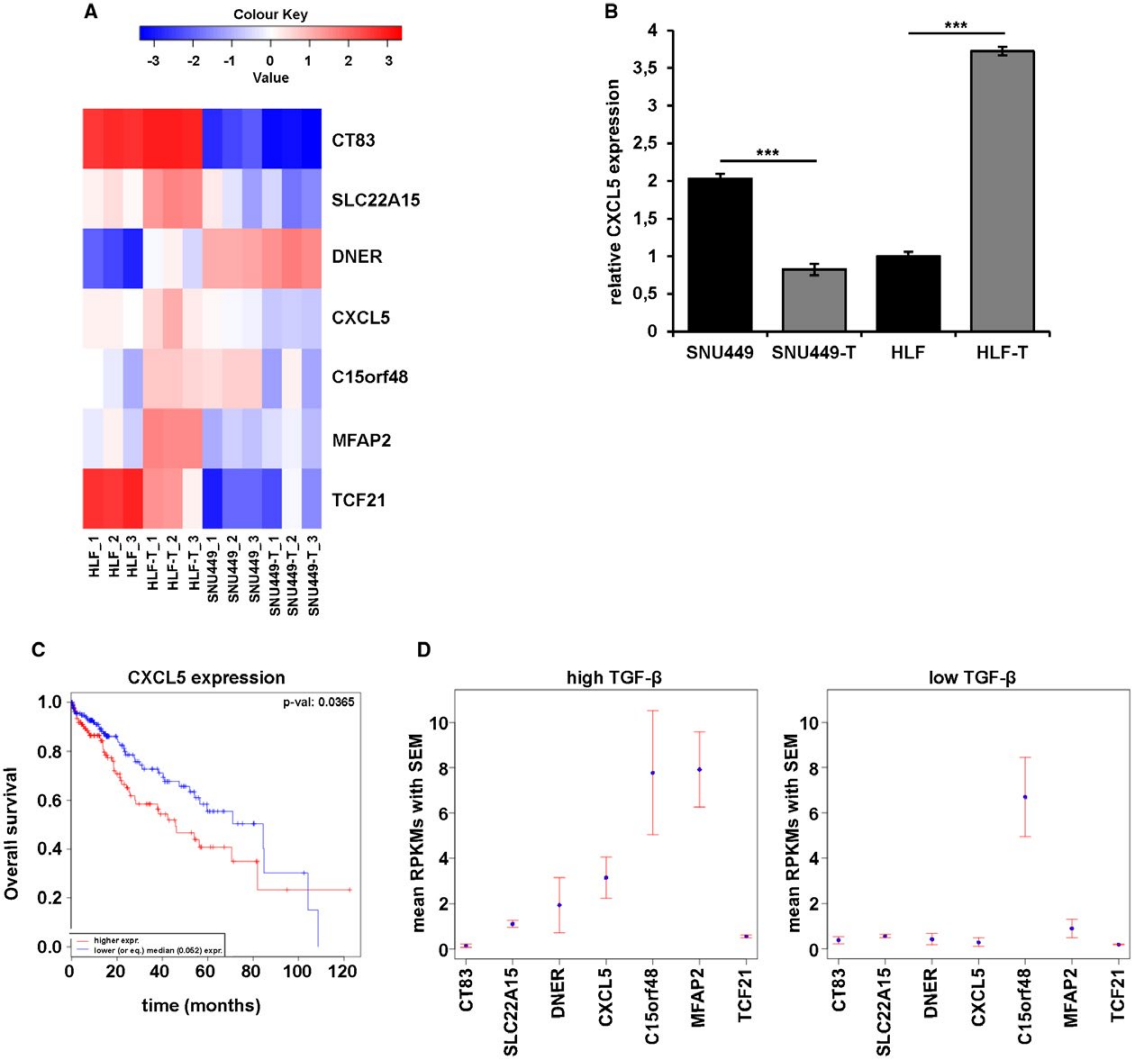
Expression profiling of genes involved in tumor‐promoting mechanisms of TGF‐β. (A) Heat map of selected target genes. (B) qPCR validation of CXCL5 expression in SNU449/SNU449‐T and HLF/HLF‐T cells. (C) Kaplan‐Meier survival curves showing higher (red) or lower levels (blue) of CXCL5 expression and corresponding overall survival in 360 HCC patients from TCGA RNAseqV2. (D) RPKM values of the selected target genes in high TGF‐β (RPKM > 20, left panel) and low TGF‐β‐expressing samples (RPKM < 5, right panel). Data are expressed as mean ± SD. Error bars depict SD from at least three individual experiments. ***P < 0.001. Abbreviations: RPKM, mean reads per kilobase per million mapped reads; qPCR, quantitative reverse‐transcriptase polymerase chain reaction; TCGA, the Cancer Genome Atlas.

To identify tumor‐promoting mechanisms of TGF‐β, we determined differential changes in gene expression of the opposing HCC cell models with and without long‐term TGF‐β treatment. Differences in the transcriptome profile were assessed by whole genome microarrays, which revealed 932 genes that were differentially expressed by exhibiting a 2‐fold upregulation or downregulation (Supporting Fig. S2). To filter genes relevant in HCC patients, we correlated expression data with information on HCC patient survival from the Cancer Genome Atlas platform. We identified six upregulated and one downregulated target genes that exclusively showed expression in TGF‐β‐positive HCC patients, among them CXCL5 (Fig. 4A, Supporting Fig. S2). The expression of CXCL5 and other selected target genes were verified by real‐time quantitative PCR analysis in SNU449/SNU449‐T and HLF/HLF‐T cells (Fig. 4B, Supporting Fig. S3). Most notably, levels of CXCL5 expression decreased when SNU449 cells were treated long‐term with TGF‐β, whereas levels of CXCL5 increased in HLF cells under these conditions. CXCL5 expression depended on TGF‐β signaling in HLF‐T cells, whereas CXCL5 expression remained unaffected by TGF‐β interference in SNU449‐T cells (Supporting Fig. S4). Overall survival analysis showed that patients with high levels of CXCL5 have a significantly shorter overall survival (Fig. 4C). Target genes such as C15orf48, CT83, DNER, MFAP2, and SLC22A15 also showed a lower overall survival when highly expressed in patients (Supporting Fig. S5). In contrast, TCF21 displayed a different pattern as patients with low expression correlated with reduced overall survival, as suggested by the cellular HCC model. Importantly, upregulated and downregulated target genes correlated with high (mean reads per kilobase per million mapped reads [RPKM] > 20) and low (RPKM < 5) expression of TGF‐β in HCC patients, respectively (Fig. 4D). Together, expression profiling of SNU449 and HLF cells subjected to long‐term TGF‐β treatment identified target genes that might play an important role in pro‐oncogenic functions of TGF‐β in HCC.

### CXCL5 Expression is Dependent on TGF‐β and Affects Cell Invasion and Tumor Formation

**Figure 5:**
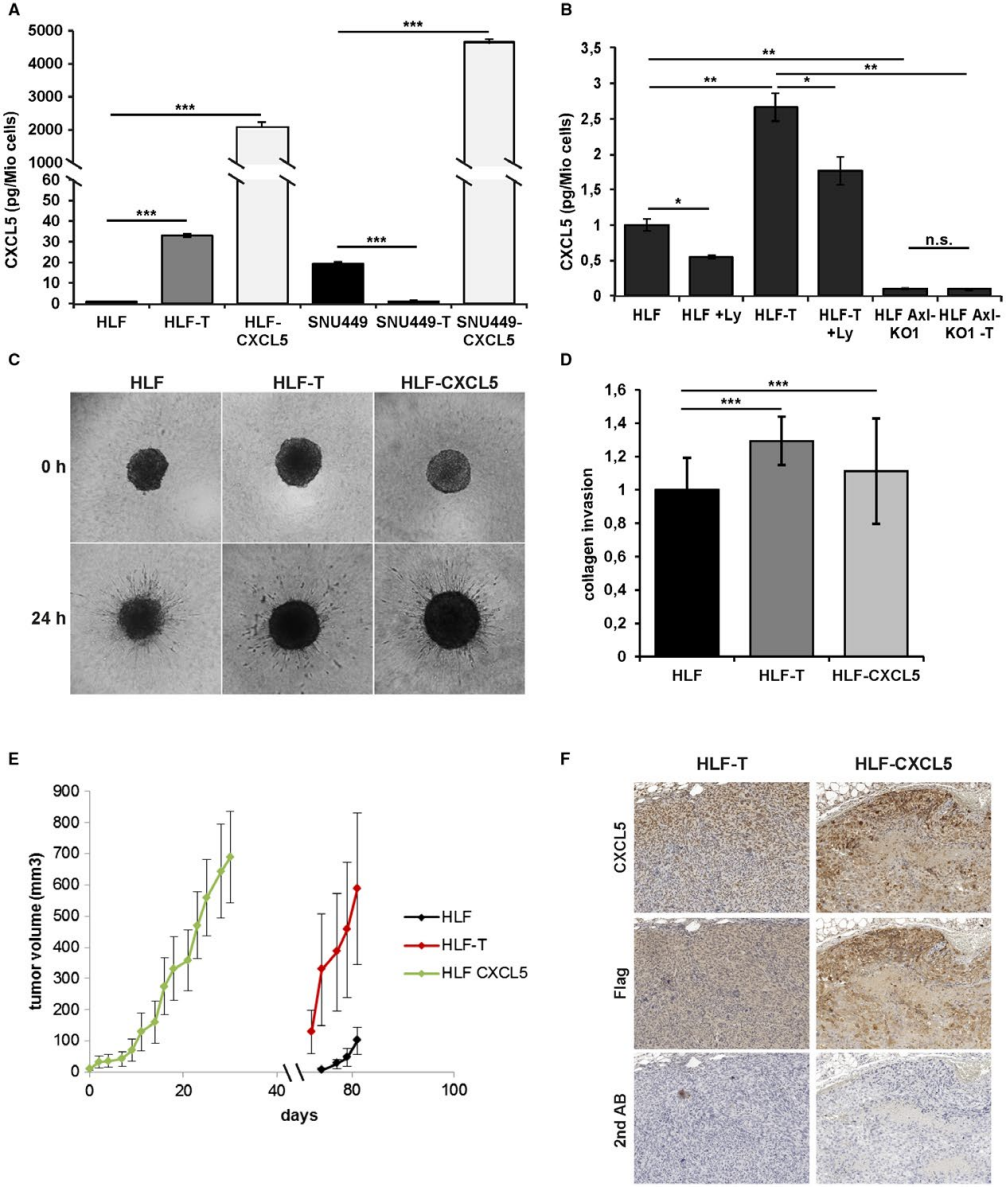
Regulation of CXCL5 and its role in cell invasion and tumor formation. (A) CXCL5 secretion of cells was assessed by ELISA. (B) CXCL5 secretion in HLF/HLF‐T cells and those treated with 10 µM Ly for 72 hours, as well as in HLF‐Axl‐KO1 and HLF‐Axl‐KO1‐T cells. (C) Representative images of hepatospheres consisting of HLF/HLF‐T/HLF‐CXCL5 cells. (D) Quantitative analyses of respective hepatosphere invasion into collagen gels. (E) Volumes of HLF/HLF‐T/HLF‐CXCL5‐derived tumors. (F) Immunohistochemical analysis showing consecutive tumor sections of HLF‐T‐derived and HLF‐CXCL5‐derived tumors stained with anti‐CXCL5 or anti‐Flag antibody. The secondary antibody was used only as control. Error bars depict SD from three individual experiments carried out in triplicates. *P < 0.05, **P < 0.01, ***P < 0.001. Abbreviations: ELISA, enzyme‐linked immunosorbent assay; n.s., not significant.

We focused on the role of CXCL5 in HCC and evaluated its effects in cells ectopically expressing CXCL5, termed HLF‐CXCL5 and SNU449‐CXCL5. We found a strong increase of secreted CXCL5 in HLF‐T and HLF‐CXCL5 in comparison to HLF cells with a 33‐fold and 2000‐fold increase, respectively (Fig. 5A). In contrast, SNU449‐T cells showed a 19‐fold decrease and SNU449‐CXCL5 cells a 4600‐fold increase of secreted CXCL5 in comparison with untreated SNU449 cells. To analyze the TGF‐β‐dependence on CXCL5, HLF and HLF‐T cells were treated with Ly, and CXCL5 secretion was determined. Notably, levels of CXCL5 in the supernatant of HLF‐T cells were significantly reduced (Fig. 5B). Because Axl signaling is involved in shifting TGF‐β responses from tumor suppression to tumor promotion in HCC,^18^ the effect of Axl on CXCL5 secretion was investigated. Importantly, CRISPR/Cas9‐mediated Axl knockout cell lines almost completely abrogated the CXCL5 secretion (Fig. 5B). Silencing of Axl also blocked the upregulation of CXCL5 after long‐term treatment with TGF‐β (Fig. 5B). These data were confirmed in a second HLF Axl knockout cell line termed “HLF Axl‐KO2” and treated long‐term with TGF‐β (data not shown).

We next studied the effect of CXCL5 on HCC cell invasion by cultivating hepatospheres into collagen gels. Both HLF‐T and HLF‐CXCL5 cell–derived hepatospheres showed a significant increase in collagen invasion compared with parental HLF cells (Fig. 5C,D). Furthermore, cells were xenografted into immune‐deficient mice to examine their tumorigenic properties. Although SNU449 cells and their derivatives formed no tumors, HLF‐T and HLF‐CXCL5 cells exhibited efficient tumor formation in 6 of 7 mice and 7 of 7 mice, respectively (Table 1). Interestingly, HLF‐CXCL5 and HLF‐T cells formed tumors after 5 days and after 2 months, respectively, whereas parental HLF cells displayed strongly retarded tumor formation at low frequency (Fig. 5E, Table 1). Staining of tumor tissues revealed that HLF‐T‐derived tumors express lower levels of CXCL5 compared with the exogenous overexpression in HLF tumors (Fig. 5F). Importantly, immunofluorescence as well as immunohistochemical analyses showed co‐expression of CXCL5 and Axl in both HLF‐T‐derived and HLF‐CXCL5‐derived tumors (Supporting Figs. S6 and S7). From these results we conclude that CXCL5 secretion depends on TGF‐β/Axl. Furthermore, CXCL5 was identified as the major driver of HCC cell invasion and tumor formation.

**Table 1:** Tumor Formation and CXCL5 Expression after Xenografting

Cell Type	Tumor Formation	CXCL5 Expression
HLF	2/7	−
HLF‐T	6/7	+
HLF‐CXCL5	7/7	+
SNU449	−	−
SNU449‐T	−	−
SNU449‐CXCL5	−	−

### CXCL5 of Long‐Term TGF‐β Treated HLF Cells Attracts Neutrophils

**Figure 6:**
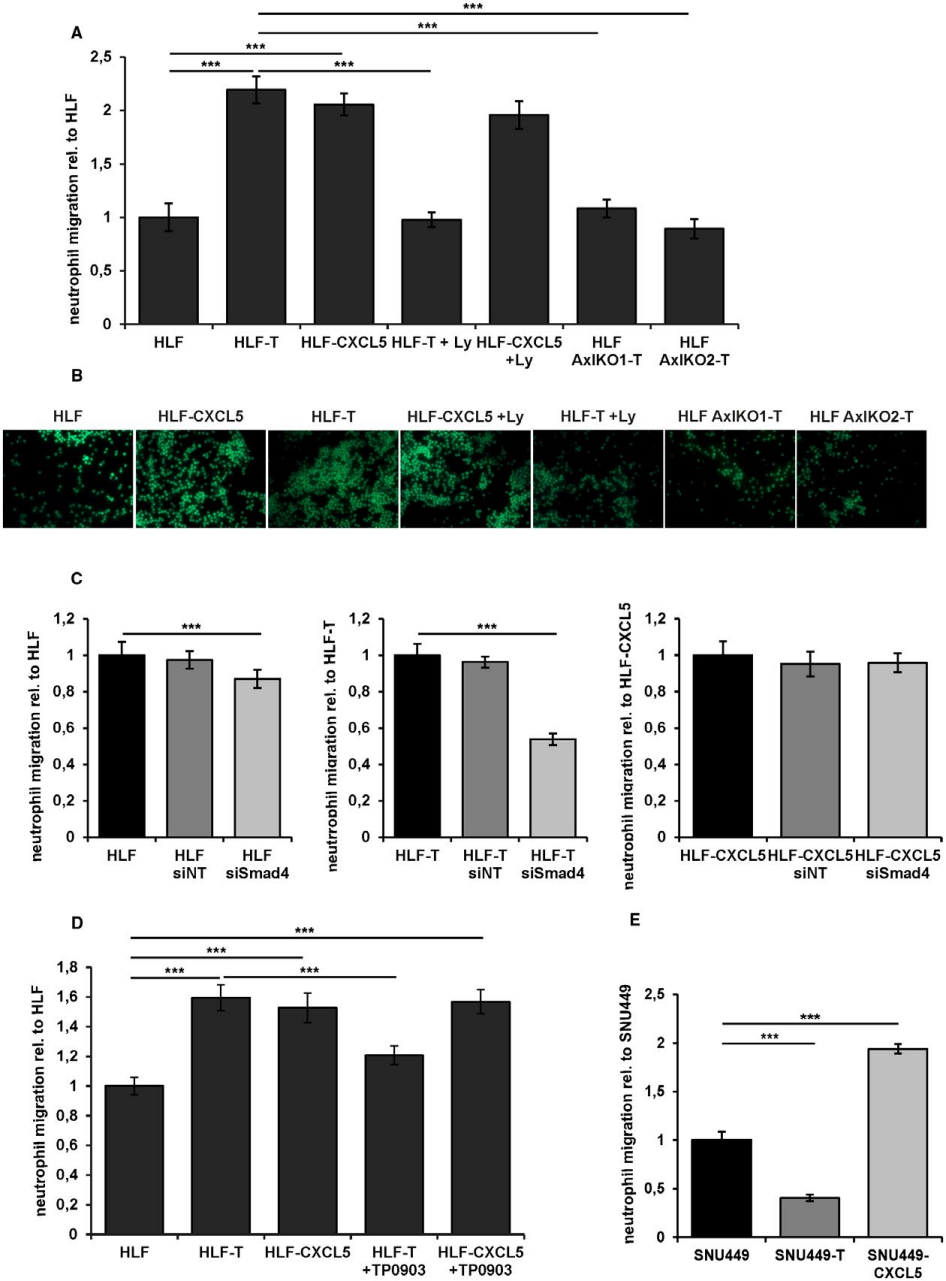
Long‐term TGF‐β treatment causes neutrophil migration. (A) Quantification of neutrophil migration as assessed by under‐agarose assay. Cell Tracker green‐labeled neutrophils were exposed to supernatants of HLF, HLF‐T, and HLF‐CXCL5 cells, those treated with 10 µM Ly for 24 hours, and long‐term TGF‐β‐treated (>10 days) HLF‐Axl‐KO1‐T and HLF‐Axl‐KO2‐T cells. (B) Representative immunofluorescence images of the under‐agarose assay shown in (A). (C) Quantification of neutrophil migration after exposure to supernatants from HLF (left panel), HLF‐T (middle panel), and HLF‐CXCL5 cells (right panel) treated with siNT or siSmad4. (D) Quantification of neutrophil migration after exposure to supernatants of HLF, HLF‐T, and HLF‐CXCL5 cells and those treated with 1 µM Axl inhibitor TP0903 for 48 hours. (E) Quantification of neutrophil migration after exposure to supernatants of SNU449, SNU449‐T, and SNU449‐CXCL5. Data are expressed as mean ± SD. ***P < 0.001.

As CXCL5 has a direct chemoattractant effect on neutrophils, we investigated the effect of long‐term TGF‐β treatment, ectopic CXCL5 expression, TGF‐β inhibition, and knockout of Axl on neutrophil migration. Interestingly, supernatants of both HLF‐T and HLF‐CXCL5 cells induced a 2‐fold increase in neutrophil migration as compared with parental HLF cells (Fig. 6A,B). Ly‐treated HLF‐T cells showed a reduction of neutrophil migration to the level of HLF cells, whereas HLF‐CXCL5 cells were unaffected by the TGF‐β inhibitor. Axl deficiency inhibited the effect of long‐term TGF‐β treatment in HLF cells. HLF AxlKO1 and HLF AxlKO2 cells that were long‐term treated with TGF‐β failed to increase neutrophil migration (Fig. 6A,B). Parental HLF, HLF‐T, and HLF‐CXCL5 cells were further treated with small interfering (si)Smad4 to evaluate the effect of TGF‐β on neutrophil migration. The Smad4 knockdown significantly reduced neutrophil migration in HLF and HLF‐T cells with 14% and 57%, respectively (Fig. 6C; left and middle panel), whereas the knockdown of Smad4 did not affect neutrophil attraction to HLF‐CXCL5 cells (Fig. 6C; right panel). Comparably, HLF‐T cells treated with the Axl inhibitor TP0903 led to reduced neutrophil migration, whereas no change was detected in TP0903‐treated HLF‐CXCL5 cells (Fig. 6D).

Contrary to the observations in HLF cells, SNU449‐T cells showed a 60% reduction in neutrophil migration compared to parental SNU449 cells (Fig. 6E). As expected, ectopic CXCL5 expression in SNU449‐CXCL5 cells enhanced neutrophil migration. Together, these data provided evidence that CXCL5 secretion of long‐term TGF‐β‐treated HLF cells increases the attraction of neutrophils. Accordingly, genetic or pharmacological intervention with either TGF‐β or Axl signaling abrogates neutrophil migration.

### Expression of CXCL5 Correlates with Advanced Tumor Stages and Neutrophil Infiltration in HCC Patients

**Figure 7:**
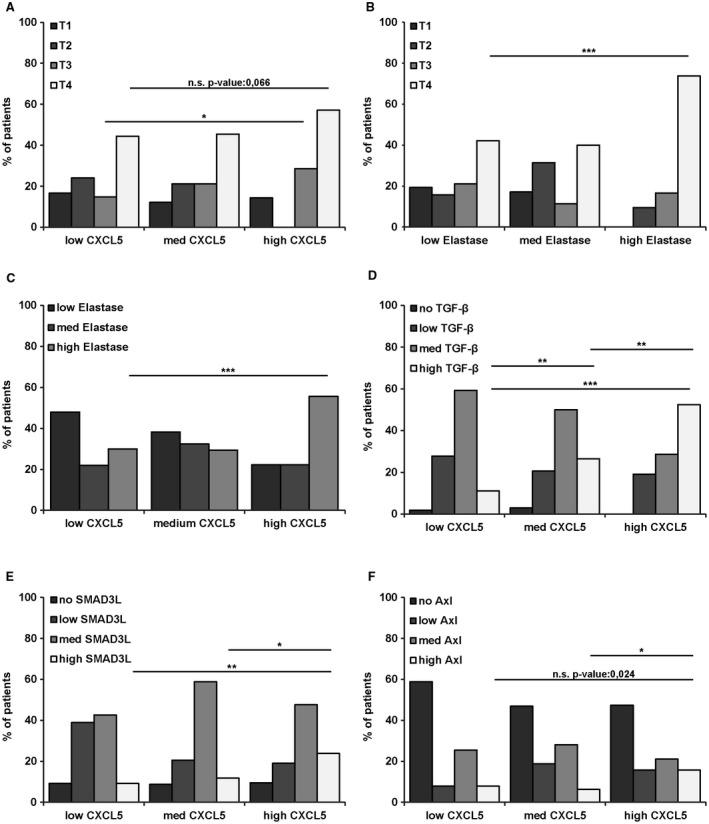
Correlation of CXCL5 with tumor staging and neutrophil attraction as well as TGF‐β and Axl expression in HCC patient samples. Immunohistochemical staining intensities of CXCL5 and elastase were scored with low, medium, and high protein levels, whereas TGF‐β, Axl, and Smad3L were scored with no, low, medium, and high. (A) Correlation of CXCL5 expression with tumor stages. (B) Correlation of elastase with tumor stages. (C‐F) Correlation of CXCL5 expression with elastase (C), TGF‐β1 (D), Smad3L (E), and Axl expression (F). Data are expressed as mean ± SD. *P < 0.05; **P < 0.01; ***P < 0.001. Abbreviation: n.s., not significant.

We immunohistochemically determined the levels of CXCL5, elastase, TGF‐β, Axl, and Smad3L phosphorylation in HCC patient samples (n = 133) to assess the clinical relevance. Patients expressing high levels of CXCL5 as well as high levels of elastase exhibited more advanced tumor stages (Fig. 7A,B; Supporting Fig. S8). Accordingly, high CXCL5 levels correlated with high elastase levels that reflect the infiltration of neutrophils (Fig. 7C). CXCL5 showed no correlation with HCC patient records such as age, gender, hepatitis virus infection or cirrhosis (Supporting Table S2), whereas medium and high elastase expression showed a significant association with the hepatitis B virus status (Supporting Table S3). Furthermore, CXCL5 expression strongly correlated with TGF‐β expression, Smad3L phosphorylation, and Axl expression (Fig. 7D‐F), confirming our results in cellular models and suggesting that CXCL5 is regulated through the TGF‐β/Smad3/Axl signaling axis. Overall survival was not significantly affected by the expression of CXCL5 or elastase in univariate and multivariate analyses (Supporting Table S4). High levels of elastase were associated with decreased survival in this patient cohort, albeit without statistical significance (*P* = 0.0589) (Supporting Fig. S9). Notably, medium to high levels of elastase were accompanied by an elevated recurrence status of HCC patients in univariate and multivariate analyses (Supporting Table S4). In conclusion, the expression of CXCL5 is linked to the tissue recruitment of neutrophils in HCC patients and associates with activated TGF‐β/Smad3 signaling and Axl expression.

## Discussion

TGF‐β exhibits a dual role in HCC by acting tumor‐suppressive at early stages and tumor‐promoting at later stages.^22^ Using long‐term TGF‐β‐treated HCC cell models, we aimed at elucidating the molecular mechanisms of the “TGF‐β switch” to better understand HCC progression. Both HCC models showed reduced proliferation and clonogenic growth behavior following long‐term TGF‐β stimulation, although displayed a difference in chemosensitivity and migratory abilities. In contrast to short‐term TGF‐β‐exposed HCC cells, long‐term TGF‐β stimulation upregulated CXCL5 expression in collaboration with Axl in HLF‐T cells, whereas it failed to increase CXCL5 in SNU449‐T cells. In this line, CXCL5 positively correlated with TGF‐β and Axl expression in HCC patients. These data provide solid evidence that the effect of TGF‐β signaling strongly depends on collaborating signaling pathways, as well as on the duration and intensity of TGF‐β exposure. From these observations we conclude that HCC cells use active TGF‐β signaling differently by modulating CXCL5 expression, which crucially affects the “TGF‐β switch.”

We identified a set of TGF‐β‐dependent genes relevant for HCC progression including CT83, SLC22A15, DNER, CXCL5, C15orf48, MFAP2, and TCF21 by comparing both cellular HCC models. CT83 is a tumor antigen expressed in a variety of cancer tissues and testicular germ cells.^23^ The roles of SLC22A15, an organic ion transporter, and C15orf48 remain to be examined in cancer, albeit the latter one has been found to be overexpressed in a highly metastatic HCC cell line.^24^ DNER, a transmembrane protein that carries extracellular EGF‐like repeats, was identified as a Notch ligand that mediates cell–cell interactions that can act oncogenically and anti‐oncogenically.^25^, ^26^ MFAP2, a small component of extracellular microfibrils, was shown to bind to the Notch1 receptor.^27^ TCF21 encodes a transcription factor that acts as a tumor suppressor in head and neck squamous cell carcinomas,^28^ fitting with our data that shows how decreased expression of TCF21 correlates with reduced survival of HCC patients. We further suggest clinical relevance for CT83, SLC22A15, DNER, C15orf48, MFAP2 and CXCL5, as high expression levels correlate with lower HCC patient survival. Additionally, high levels of TGF‐β, Axl, and CXCL5 associate with advanced tumor stages and recruitment of neutrophils into cancer tissue of HCC patients.

CXCL5 has chemotactic and activating functions on neutrophils, primarily during acute inflammatory responses. Elevated expression of CXCL5 was found in diverse tumor entities including HCC, thereby correlating with poor prognosis of patients.^29^ Zhou et al. suggested that CXCL5 promotes HCC cell proliferation, invasion, and intratumoral neutrophil infiltration.^7^ It was further demonstrated that EGF/EGFR stimulates CXCL5 production in HCC through PI3K and MAPK signaling. Interestingly, CXCL5 is overexpressed in HCCs with high metastatic potential.^30^ Zhou et al. showed that the CXCR2/CXCL5 axis contributes to epithelial to mesenchymal transition (EMT) through activating the PI3K/Akt/GSK‐3β/Snail pathway in HCC cells.^31^ Additionally, CXCL5 is an effector of tumor‐associated neutrophils that mediate the intratumoral infiltration of macrophages and regulatory T cells by secreting CCL2 and CCL17, which enhances HCC progression and sorafenib resistance.^32^

Neutrophils are important components of the inflammatory response and have dual roles in tumor development and metastasis. In response to stimulation of different cytokines, neutrophils have the potential to polarize toward an antitumorigenic phenotype (N1) in the case of acute inflammation or toward a protumorigenic phenotype (N2) in the case of chronic inflammation.^33^ The inflammatory cytokines such as TGF‐β can induce N2 phenotype of neutrophils in bone marrow and tumor microenvironment.^34^ This changes the local tumor microenvironment and facilitates microthrombus formation through the formation of neutrophil extracellular traps (NETs),^35^ composed of DNA, histones, and antimicrobial proteins.^36^ Neutrophils are also recruited and activated by platelets, which stimulate TGF‐β release and platelet–tumor cell aggregation.^37^ NETs stimulate the intrinsic pathway of the coagulation cascade, ultimately generating thrombin and activating platelets.^38^ Axl signaling enhances platelet degranulation and aggregation responses, thus promoting platelet activation and mediating thrombus formation.^39^ Platelets also recruit and activate macrophages and neutrophils in tumor tissue, stimulating TGF‐β release and platelet–tumor cell aggregation, which prevents the lysis by natural killer cells.^37^ Additionally, they induce EMT, cell invasion, angiogenesis, and distal metastasis.^40^ Taken together, this might explain the important role of Axl/TGF‐β/CXCL5 signaling in malignant cell dissemination.

Our study revealed insights into HCC progression that could be used to better stratify patients for targeted therapy. TGF‐β‐positive patients should not be considered to be treated with Axl inhibitors, as the intervention with Axl’s anti‐inflammatory effects might enhance inflammation and tumor progression. Therefore, our data suggest treating TGF‐β/CXCL5‐positive patients directly against CXCL5, to circumvent tumor‐promoting functions of inflammation. In conclusion, the identification of CXCL5 as a key driver in HCC progression offers an innovative therapeutic approach that directly interferes with disease progression in TGF‐β/CXCL5‐positive patients.
