# Supplementary material for: Transforming Growth Factor‐β and Axl Induce CXCL5 and Neutrophil Recruitment in Hepatocellular Carcinoma
Source: Hepatology. 2018 Dec 20;69(1):222–36. doi: 10.1002/hep.30166 (PMC6590451; doi:10.1002/hep.30166)
Supplement: Supplementary file 1 [file HEP-69-222-s001.pdf]

Supporting Material and Methods

Immunohistochemistry of HCC tissue microarrays

Tumor sections with a size of 4 µm from core biopsies arrayed in triplicates were stained with anti-CXCL5 antibody (R&D Systems, Minneapolis, USA), anti-neutrophil elastase antibody (Abcam, Cambridge, UK), anti-Axl antibody (R&D), anti-phospho-Ser213-Smad3L antibody (Abcam, Cambridge, UK) or anti-TGF-β1 antibody (Santa Cruz, Dallas, USA), each at a dilution of 1:100. Secondary antibodies were employed and visualization was performed with the vectastain ABC kit using diaminobenzidine as substrate (Vector Laboratories, Burlingame, USA) and counter staining with hematoxylin (Carl Roth, Karlsruhe, Germany). Immunohistochemical staining was assessed using the Tissue Studio v4.4.1 image analysis software (Definiens, Munich, Germany). The staining was categorized into no, low, medium or high staining and correlated to patient data. Data sets were compared using GraphPad Prism v5.01 (GraphPad Software Inc., USA). Two-sided Fisher's exact test was used to analyze categorical data and survival curves were compared with the Log-rank test.

Construction of expression vectors

The full-length cDNA encoding CXCL5 was amplified by polymerase chain reaction (PCR) from the pDONR201 plasmid (DNASU Plasmid Repository, Arizona, USA) with the forward primer 5'-CTAGCCTCGAGGTTTAAACATGAGCCTCCTGTCCAGCCG-3' and the reverse primer: 5'-TGCAGCCCGTAGTTTAAACTCAGTTTTCCTTGTTTCCACCGTCCA-3'. A second set of primers was used to add a FLAG-tag (forward primer: 5'-CTAGCCTCGAGGTTTAAACATGAGCCTCCTGTCCAGCCG-3'; reverse primer: 5'-TGCAGCCCGTAGTTTAAACTTACTTATCGTCGTCATCCTTGTAATCAGAGCCTCCACCCCCGTTTTCCTTGTTTCCACCGTC-3'). The purified CXCL5 and CXCL5-FLAG PCR products were ligated into the pWPI vector according to the manufacturer’s instructions using

the In-Fusion® cloning technology (Takara Bio, Mountain View, USA). The pWPI vector containing green fluorescent protein (GFP) was used as control.

Cell culture

The human hepatoma cell lines SNU449 and HLF were cultivated in RPMI-1640 supplemented with 10% fetal calf serum (FCS) and in DMEM plus 10% FCS, respectively. TGF-β signaling was stimulated by short-term treatment of cells for 15 minutes or 24 hours with 2.5 ng/mL TGF-β1 (R&D Systems, Minneapolis, USA), or by long-term treatment for >10 days with 1 ng/mL. To inhibit TGF-β signaling, the low molecular weight compound LY2109761 (Ly; Santa Cruz, Dallas, USA) antagonizing TGF-β receptors I/II was used at a concentration of 10 μM for 24 hours. To interfere with Axl signaling, TP0903 (MedChem Express, New Jersey, USA) was employed at a concentration of 1 μM. The pWPI vector with CXCL5 or CXCL5-FLAG and without CXCL5 were packaged into lentiviral particles for subsequent infection of SNU449 and HLF cells. Transduced cells were enriched for GFP expression by cell sorting. All cells were kept at 37°C and 5% CO2 and routinely screened for the absence of mycoplasma. The authentication of hepatoma cells was performed by short tandem repeat DNA profiling analysis.

Knockdown by RNA interference

Cells were seeded on 6-well plates and either transfected with 80 nM of non-target small interfering (si)RNA or with 120 nM of siRNA against human Smad4 (Dharmacon, Town, UK) using Oligofectamine (Life Technologies, Green Island, USA) according to the manufacturer’s instruction. Cells were processed for further analysis after 48 hours of siRNA transfection.

Transwell assay

For studying cell migration in a Transwell assay, 2.5 x 10^4 cells were resuspended in 150 µL medium containing 1% FCS and seeded on Transwell permeable supports for 24-well plates with a pore size of 8 µm (Corning, New York, USA). 700 µL of the respective culture medium containing 10% FCS was added to the bottom of the well to generate a gradient for cell migration. To detect cell movement, medium was removed and migrated cells on membranes were fixed with 4 % paraformaldehyde after 16 hours. Cell nuclei of migrated cells at the bottom side of the membrane were stained with Hoechst 33342 (Life Technologies, Green Island, USA) and counted under the fluorescence microscope (Nikon, Tokyo, Japan). To analyze cell invasion, 24-well Transwell permeable supports were coated with rat-tail collagen (BD Biosciences, NJ, USA) prior to seeding of cells. Three independent experiments were performed in triplicates.

Wound healing assay

For studying cell migration in a scratch wound assay, 1 x 10^6cells were seeded on 6-well plates and artificial wounds were inflicted to the cell layer by scratching with sterile pipette tips. Images were taken by phase contrast microscopy (Nikon, Tokyo, Japan) immediately after wounding and after 24 hours. The migrated area of cells into the wound was quantified with ImageJ software (https://imagej.nih.gov/ij/). Three independent experiments were performed for each analysis.

Determination of cell viability and inhibitory concentration $(IC)_{50}$

Cell viability was determined using the 3-(4,5 dimethylthiazol-2-yl)-2,5-diphenyltetrazolium bromide (MTT) assay as described (1). Briefly, cells were seeded in triplicates at a density of 5 x 10^3 cells per 96-well. After 24 hours, cells were incubated with drug-containing medium for 72 hours. Cells were incubated with MTT solution (5 mg/ml; Sigma, St. Louis, USA) and

medium was replaced with dimethyl sulfoxide after 5 hours. The absorbance was measured at 620 nm in a microplate reader (Asys HiTech, Salzburg, Austria). IC_{50} values were obtained by log-linear interpolation of data points and are depicted by dose-response curves using the software GraphPad Prism® 5.01. Three independent experiments were performed for each analysis.

Clonogenic survival assay

500 cells, either untreated or treated with TGF-β1, were each seeded in a 6-well plate for 24 hours and incubated with the respective standard medium for 10 days at 37°C and 5% CO_2. Colonies were fixed with methanol/acetic acid (3:1) and stained with 0.25% crystal violet. The crystal violet of fixed cells was solubilized with 1% SDS and the absorbance was photometrically determined at 560 nm. Three independent experiments were performed in triplicates.

Immunoblotting

Immunoblotting was performed as described previously (2). The primary antibodies used were anti-pSmad2 (Millipore, Darmstadt,Germany) 1:1.000, anti-Smad2/3 (BD, New Jersey, USA) 1:1.000, anti-Smad4 (Cell Signalling Technology, Beverly, USA), anti-Snail (Cell Signalling Technology, Beverly, USA) 1:1.000 and anti-β-actin (Sigma, St. Loius, USA) 1:2.500. Horseradish peroxidase-conjugated secondary antibodies (Calbiochem, LaJolla, USA) were used at dilutions of 1:10.000.

Immunofluorescence analysis

Cells were seeded onto collagen-coated glass slides at a confluency of 80%. Subsequently, cells were washed with phosphate-buffered saline (PBS), permeabilized with 0.05 % Triton X-100 and fixed with 4% paraformaldehyde. After blocking with 5% bovine serum

albumin/PBS, slides were incubated with anti-Smad2/3 antibody (BD, New Jersey, USA) at a dilution of 1:100 or Texas Red-X phalloidin (Invitrogen, Carlsbad, USA) at a dilution of 1:500. The corresponding secondary antibody (Invitrogen, Carlsbad, USA) was applied after 1 hour. Nuclei were visualized with 4',6-diamidino-2-phenylindole (DAPI; Sigma, St. Loius, USA) at a dilution of 1:1.000. Imaging was performed by confocal immunofluorescence microscopy (Zeiss, Jena, Germany).

Proliferation kinetics

5 x 10^4 cells were seeded onto 6-well plates and cell numbers were determined after 72 hours with a multichannel cell analyzer (CASY; Schärfe Systems, Reutlingen, Germany). Three independent experiments were performed in triplicates.

Enzyme-linked immunosorbent assay (ELISA)

The ELISA for CXCL5 (R&D Systems, Minneapolis, USA) was carried out according to manufacturer’s protocols. Briefly, CXCL5 expression was assessed in whole cell lysates by adjusting the total protein concentration to 100 µg/mL before carrying out the ELISA. CXCL5 secretion into cell supernatants was assessed after cultivation of cells in serum-free medium for 24 hours. A seven-point standard curve was generated for every plate and quantification was performed using the GraphPad Prism 5.0 software (GraphPad Software, La Jolla, USA). All samples were measured in biological replicates and technical triplicates.

Formation of hepatospheres and invasion into collagen gels

Hepatospheres were formed using the GravityPLUS™ hanging drop system according to the protocol of the manufacturer (InSphero AG, Schlieren, Switzerland). HLF, HLF-T and HLF-CXCL5 cells were used in an amount of 2.000, 3.000 and 1.000 cells, respectively. After 96 hours, the formed hepatospheres were imbedded into rat-tail collagen, diluted 1:5 with

DMEM plus 10% FCS. Relative invasion depth of the spheroids into the collagen was assessed by making 4-fold magnified images of the embedded spheroids at 0 hour and 24 hours and measuring their relative hepatosphere size via ImageJ software (https://imagej.nih.gov/ij/).

Isolation of neutrophils

Neutrophils were isolated using standard techniques (3). Bone marrow cells were isolated by flushing femur and tibia of C57BL/6 mice with RPMI-1640 supplemented with 2 mM EDTA. Erythrocytes were removed using hypotonic lysis in 0.2% NaCl for 20 seconds at room temperature, followed by addition of 1.6% NaCl resulting in hypertonic rescue. Neutrophils were purified using Histopaque density centrifugation (Sigma, St. Loius, USA). Purified neutrophils were resuspended in RPMI-1640 plus 10% heat-inactivated FCS at a concentration of 5×10^6 cells/mL. Neutrophils were labeled with 5 µM Cell Tracker™ Green CMFDA (Invitrogen, Carlsbad, USA) in PBS for 45 minutes by incubation at 37°C and 5% CO_2. Cells were then washed with RPMI 1640 plus 10% FCS and diluted for further use.

Under agarose assay

The under agarose assay was performed as previously described (4, 5) with minor modifications. 60 mm culture dishes were filled with 6 ml of a 0.6% agarose solution containing 50% H_2CO_3-buffered Hank's balanced salt solution and 50% RPMI-1640 culture medium with 20% heat-inactivated FCS. After the agarose solidified, three wells were cut into the gel along a straight line using a steel punch guided by a template, each with a diameter of 4 mm and 1.8 mm apart. The gels were allowed to equilibrate for 1 hour by incubation at 37°C and 5% CO_2. The center well was loaded with 20 µl of purified neutrophils (5×10^6 cells/mL), and the outer wells were loaded with 20 µl of conditioned medium. Supernatants were harvested from 3.5×10^6 cells. The loaded gels were incubated for 2 hours at 37°C and

5% CO_2. Images were taken on an inverted phase contrast microscope (Nikon, Tokyo, Japan). Two target areas of 2 mm x 2 mm were analyzed. Cell numbers were determined by Image J (6).

CRISPR/Cas9-mediated genomic editing of the AXL locus

The *AXL* gene was disrupted in hepatoma cell lines using the human Axl gRNA CRISPR lentivirus set (K0161411, ABM, Milton, Ontario, Canada) as described recently (7). Two established knockout cell lines, i.e. HLF-Axl-KO1 and HLF-Axl-KO2, of the human hepatoma HLF cells were used in this study.

Transcriptome profiling using microarrays

Total RNA was isolated from triplicates of SNU449, SNU449-T, HLF and HLF-T cells using the RNeasy Mini kit (Qiagen, Hilden, Germany). The integrity and quantity of RNA was analyzed using the Agilent Bioanalyzer (Agilent, Santa Clara, USA). cDNA labeling was performed using the low-input QuickAmp labeling kit and hybridization on human SurePrint G3 8x60K pangenomic microarrays (Agilent, Santa Clara, USA) as well as scanning of signal intensities were performed according to the manufacturer's protocol.

Bioinformatic analysis

Expression data were analyzed with the R/Bioconductor statistical computing environment (http://www.r-project.org, http://www.bioconductor.org). Raw expression data exported from Agilents' Feature Extraction Software were imported and processed by background correction and quantile normalization using the bioconductors LIMMA package. In order to exclude unexpressed genes from the analysis, the data were filtered on the Agilent flag "IsWellAboveBG". Only probe sets with a log2 intensity, where at least 100 percent of samples in any 1 out of 4 conditions have flags, were retained for the analysis of differentially

expressed genes, keeping 27.859 probes. Hierarchical cluster analysis was performed using R's 'hclust' function using the Euclidean distance matrices and a complete linkage clustering of normalized expression values. Principal component analysis was performed with normalized expression values using the R function 'prcomp', and the first two principal components were plotted. For detection of differentially expressed genes, a linear model was fitted to the microarrays, and an empirical Bayes moderated paired T-test was performed using the "limma" package from Bioconductor. Raw p-values were adjusted for multiple testing using the Benjamini-Hochberg procedure. Transcripts exhibiting a 2-fold up- or downregulation (FC > 2 or < -2) and an adjusted p-value < 0.05 were regarded as differentially expressed.

The Cancer Genome Atlas (TCGA) data analysis

The Level 3 TCGA RNASeq and RNASeq version2 data for HCC were downloaded from the TCGA Data Portal (https://tcga-data.nci.nih.gov/tcga/tcgaHome2.jsp). For RNASeq, 26 samples containing clinical patient information were available, consisting of nine solid tissue normal samples (TCGA sample type 11) and 17 primary solid tumor samples (TCGA sample type 01). For RNASeqV2, 410 samples with clinical patient information were available, including 50 normal solid tissues and 360 primary solid tumor samples. Raw counts from gene level data were used for analysis of differential expressed genes using Bioconductors "edgeR" package: Gene counts were normalized using edgeR's TMM (trimmed mean of M values). Differential gene expression between tumor- and normal samples of RNASeq- and RNASeqV2 sample data, respectively, was assessed using edgeR's exact test.

Survival curves

Survival analysis was performed in R using the "survival" package. Therefore, RPKM (Mean Reads Per Kilobase per Million mapped reads) values were calculated from TMM-normalized

counts using edgeR‘s RPKM function. In order to split the patients into two groups with different survival probabilities exhibiting higher or lower gene expression, the median-div approach was used. For each gene, the patients were split into high- or low-expressing groups according to whether the expression of the candidate gene was greater than the median expression of the candidate gene

Quantitative reverse-transcriptase polymerase chain reaction (qPCR)

RNA was extracted, DNaseI treated and reverse transcribed using a RNA isolation and cDNA synthesis kit (Quiagen, Hilden, Germany) as recommended by the manufacturer. Aliquotes of the cDNA were employed for Fast SYBR green qPCR (Applied Biosystems, Foster City, USA) and quantified with the 7500 Fast Real-Time PCR System (Applied Biosystems, Foster City, USA). Primer sequences are shown in Supporting Table 1.

Xenograft tumor formation

A total of 5×10^6 human hepatoma cells were subcutaneously injected into the flanks of immunodeficient SCID mice (C.B-17/Prkcdscid; Charles River Laboratories, Sulzfeld, Germany). Tumor volume was determined as described recently (8). All experiments were performed according to the Austrian guidelines for animal care and protection.

Statistical analysis

Data were expressed as means ± standard deviation (SD) or standard error of the mean (SEM). The statistical significance of differences was evaluated using an unpaired, non-parametric Student’s t-test. Significant differences between experimental groups were * $p<0.05$, ** $p<0.01$ or *** $p<0.005$.

References to Supporting Material and Methods

1. van Zijl F, Mall S, Machat G, Pirker C, Zeillinger R, Weinhaeusel A, Bilban M, et al. A human model of epithelial to mesenchymal transition to monitor drug efficacy in hepatocellular carcinoma progression. Mol Cancer Ther 2011;10:850-860.
2. Gotzmann J, Huber H, Thallinger C, Wolschek M, Jansen B, Schulte-Hermann R, Beug H, et al. Hepatocytes convert to a fibroblastoid phenotype through the cooperation of TGF-beta1 and Ha-Ras: steps towards invasiveness. J Cell Sci 2002;115:1189-1202.
3. Swamydas M, Lionakis M. Isolation, Purification and Labeling of Mouse Bone Marrow Neutrophils for Functional Studies and Adoptive Transfer Experiments, 2013.
4. Nelson RD, Quie PG, Simmons RL. Chemotaxis under agarose: a new and simple method for measuring chemotaxis and spontaneous migration of human polymorphonuclear leukocytes and monocytes. J Immunol 1975;115:1650-1656.
5. Foxman EF, Kunkel EJ, Butcher EC. Integrating conflicting chemotactic signals. The role of memory in leukocyte navigation. J Cell Biol 1999;147:577-588.
6. Heit B, Kubes P. Measuring chemotaxis and chemokinesis: the under-agarose cell migration assay. Sci STKE 2003;2003:Pl5.
7. Scharf I, Bierbaumer L, Huber H, Wittmann P, Haider C, Pirker C, Berger W, et al. Dynamics of CRISPR/Cas9-mediated genomic editing of the AXL locus in hepatocellular carcinoma cells. Oncol Lett 2018;15:2441-2450.
8. Reichl P, Dengler M, van Zijl F, Huber H, Fuhrlinger G, Reichel C, Sieghart W, et al. Axl activates autocrine transforming growth factor-beta signaling in hepatocellular carcinoma. Hepatology 2015;61:930-941.

Supporting Figure Legends

Supporting Fig. S1

Representative images of wound healing assays of SNU449/SNU449-T (left panel) and HLF/HLF-T cells (right panel). The images were taken immediately after the scratch was performed (0 h) and after 24 hours (24 h). The black lines indicate the borders between cell and cell-free areas.

Supporting Fig. S2

Expression profiling of genes involved in tumor-promoting mechanisms of TGF-β. (A) Schematic workflow of gene expression analysis and subsequent target gene selection. (B) Data clustering of the preprocessed and filtered gene expression results using the principal component analysis.

Supporting Fig. S3

qPCR analyses of (A) C15orf38, (B) CT83, (C) DNER, (D) MFAP2, (E) SLC22A15, and (F) TCF2 expression levels in SNU449/SNUN449-T cells and HLF/HLF-T cells. Error bars depict SD from at least 3 individual experiments that were carried out in triplicates. Statistical significance is indicated with asterisks (**P<0.01, ***P < 0.001). Abbreviations: n.s., not significant; qPCR, quantitative reverse-transcriptase polymerase chain reaction; SD, standard deviation.

Supporting Fig. S4

CXCL5 expression depends on TGF-β signaling in HLF-T cells. (A) SNU449/SNU449-T and (B) HLF/HLF-T cells were treated with 10 μM TGF-β inhibitor LY2109761 (Ly) for 24 hours and analyzed for CXCL5 expression by qPCR. Data are expressed as mean ± SD. Error bars depict SD from at least 3 individual experiments. Statistical significance is indicated with asterisks (*P<0.05, **P<0.01, ***P < 0.001). Abbreviations: n.s., not significant; SD, standard deviation.

Supporting Fig. S5

Kaplan-Meier survival curves showing higher (red) or lower levels (blue) of (A) C15orf48, (B) CT83, (C) DNER, (D) MFAP2, (E) SLC22A15, (F) TCF21 expression and corresponding overall survival in 360 HCC patients. Data were obtained from TCGA RNAseqV2.

Supporting Fig. S6

Co-expression of Axl and CXCL5 in GFP-tracked HLF-T and HLF-CXCL5-derived tumors as analyzed by immunofluorescence microscopy. (A) Confocal immunofluorescence analysis of GFP (green), CXCL5 (red) and Axl expression (yellow) in HLF-T tumors. The right panel represents a merge of CXCL5 and Axl expression (orange). Cell nuclei were counterstained with DAPI (blue). (B) Immunofluorescence analysis of HLF-CXCL5-derived tumors as described in (A).

Supporting Fig. S7

Co-expression of Axl and CXCL5 in HLF-T and HLF-CXCL5-derived tumors as analyzed by immunohistochemistry. (A) Immunohistochemical analysis showing consecutive tumor sections of HLF-T-derived tumors stained with anti-CXCL5 or anti-Axl antibody. Red boxes in upper panels show location of fivefold magnified regions in lower panels. (B) Immunohistochemical analysis of HLF-CXCL5-derived tumors as described in (A).

Supporting Fig. S8

Representative images of HCC patient samples immunohistochemically stained for (A) CXCL5 and (B) elastase. Immunohistological staining intensity of CXCL5 was assessed by Definiens® Tissue Studio v4.4.1. image analysis software. Low, medium (Med) and high CXCL5 staining was calculated by < 24.75%, 24.75%-29% and > 29% of relative diaminobenzidine staining intensity per tissue microarray triplicate. Low, medium (Med) and high elastase staining was calculated by <1, 1-3, >3 neutrophils per tissue microarray triplicate. Red arrows indicate elastase-positive neutrophils.

Supporting Fig. S9

Kaplan-Meier survival analyses of HCC patients expressing low, medium and high levels of elastase.

Haider et al, Supporting Figure S1

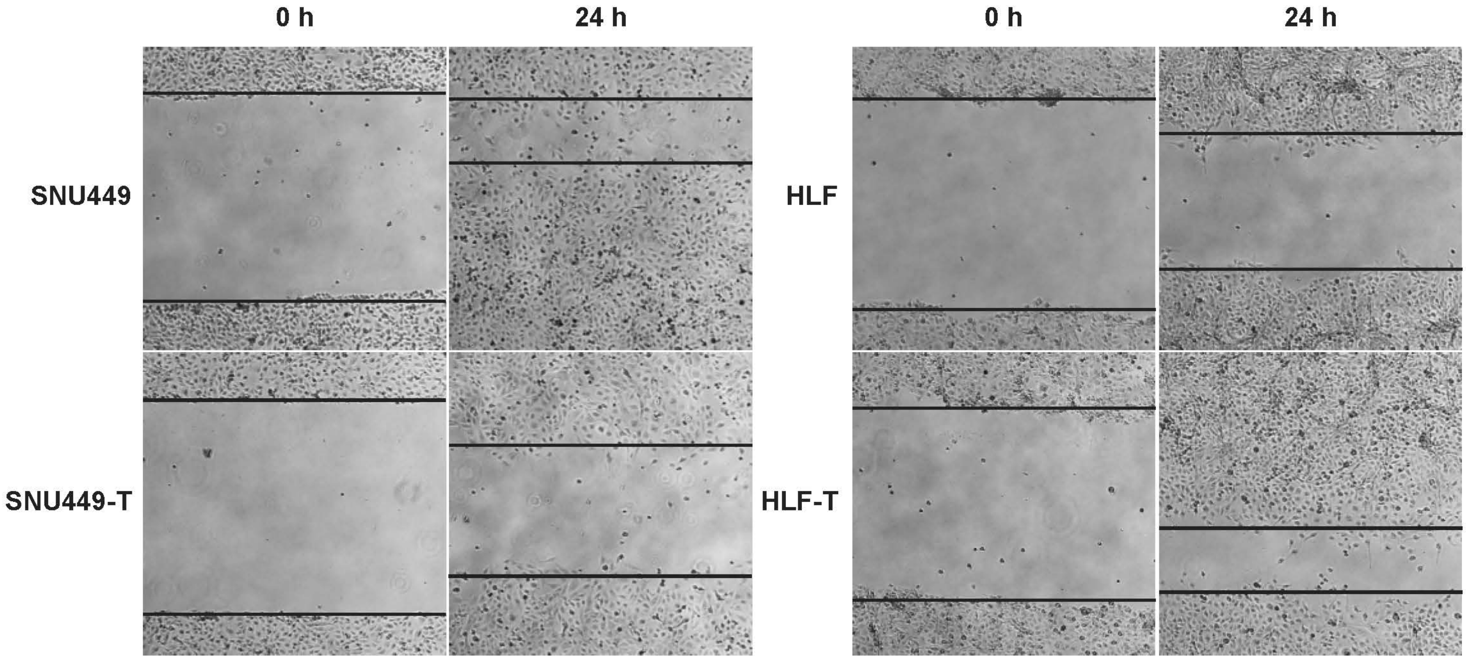

Haider et al, Supporting Figure S2

A

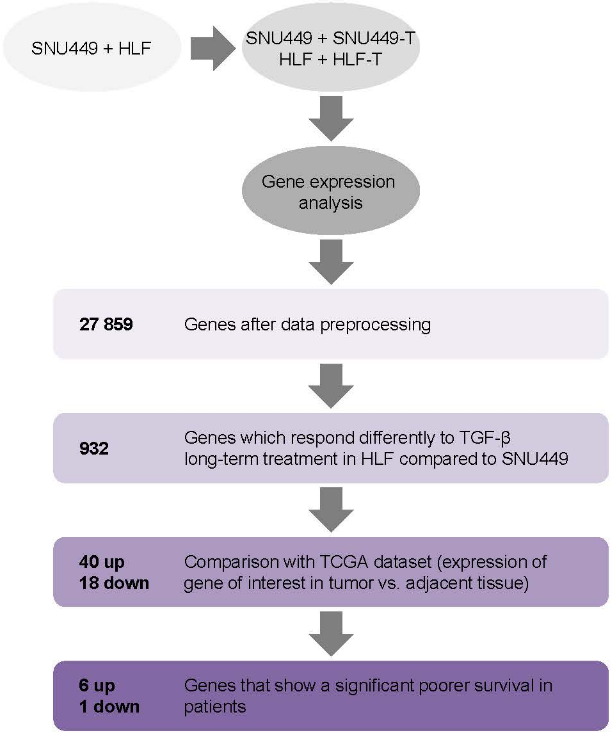

B

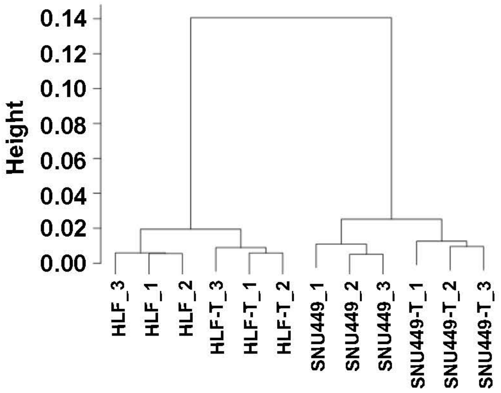

Haider et al, Supporting Figure S3

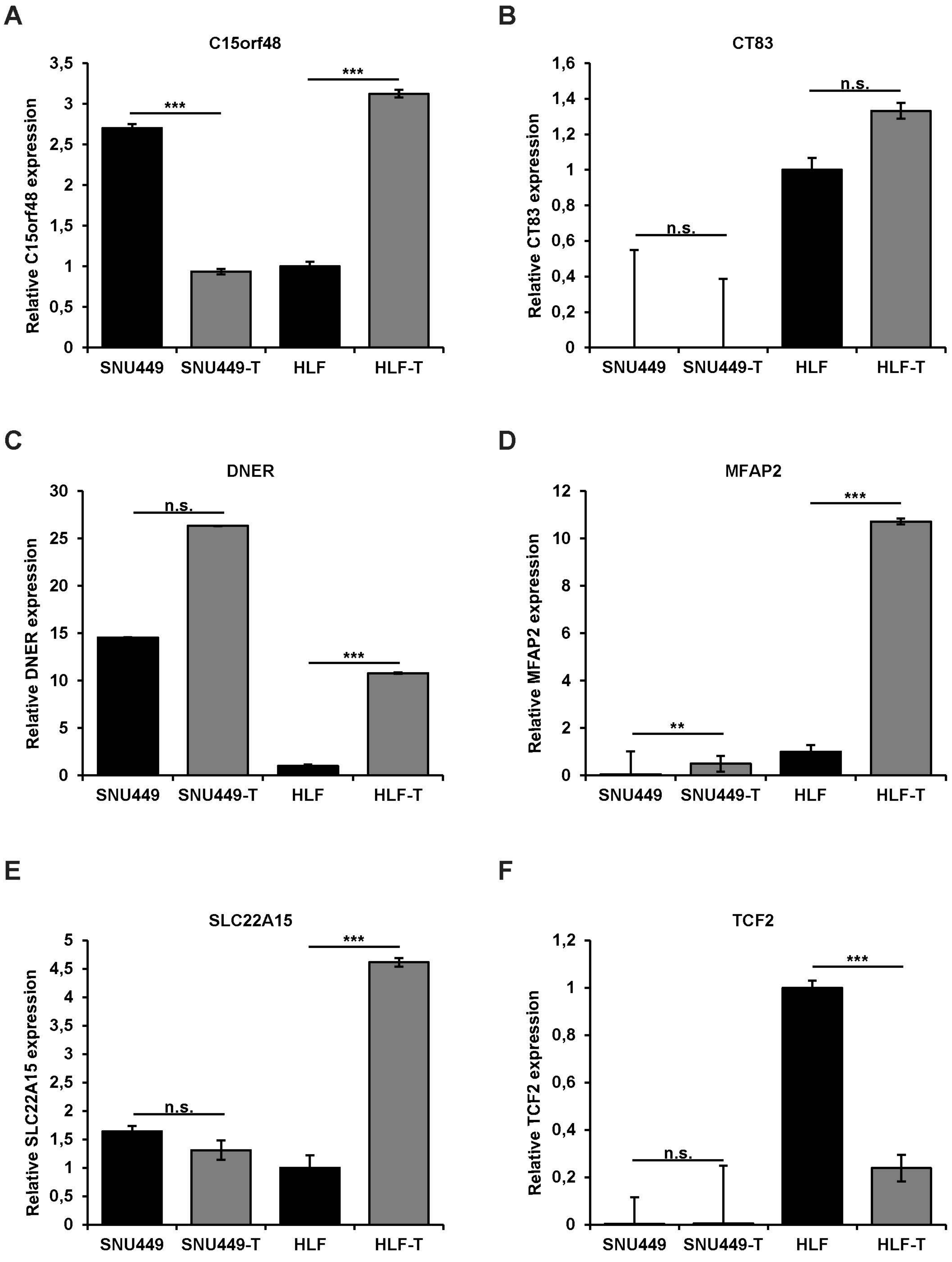

Haider et al, Supporting Figure S4

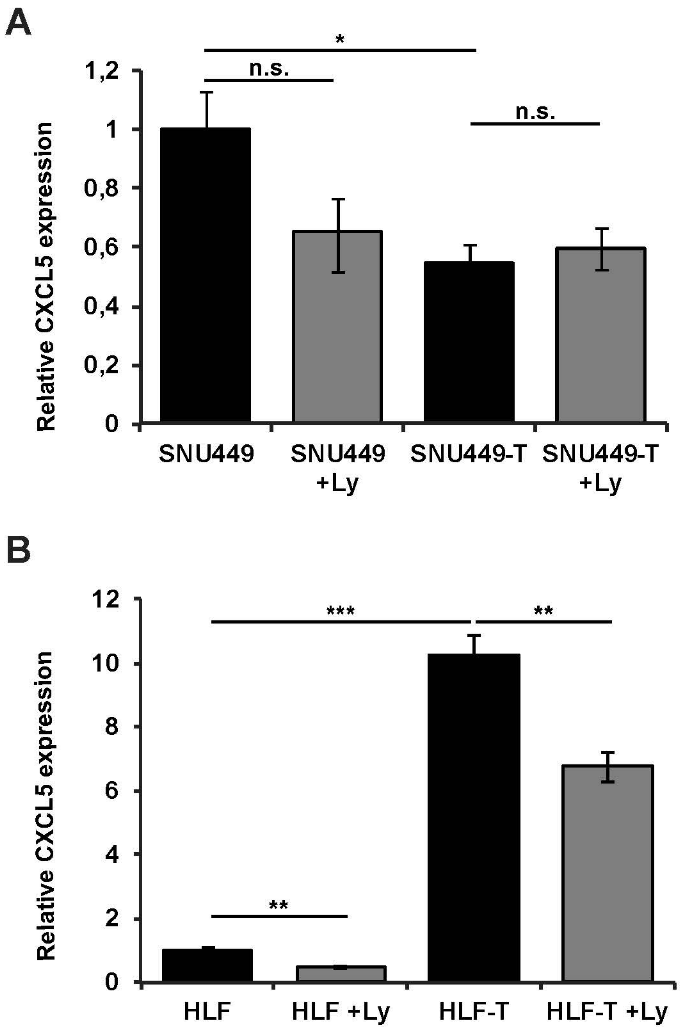

Haider et al, Supporting Figure S5

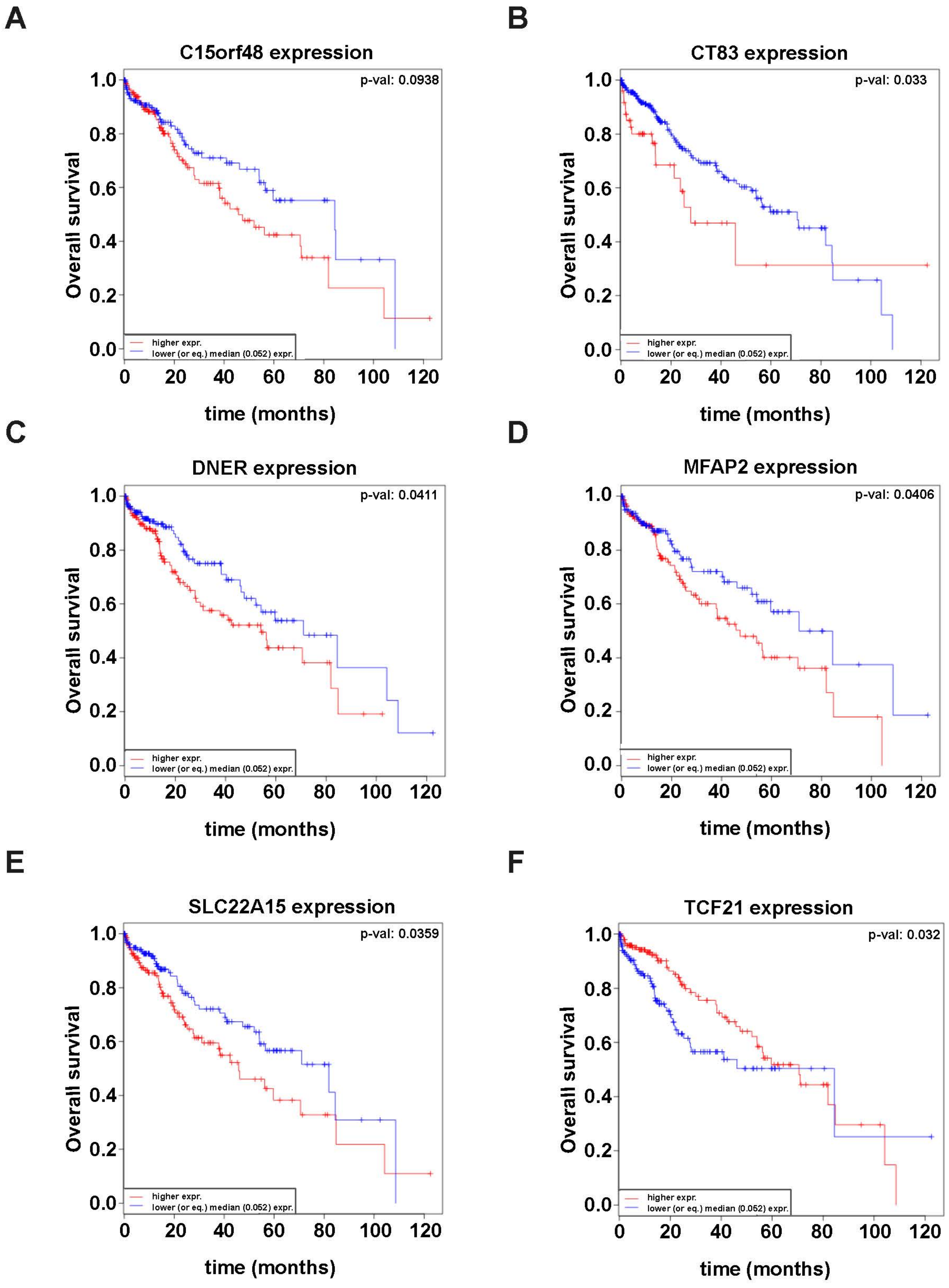

Haider et al, Supporting Figure S6

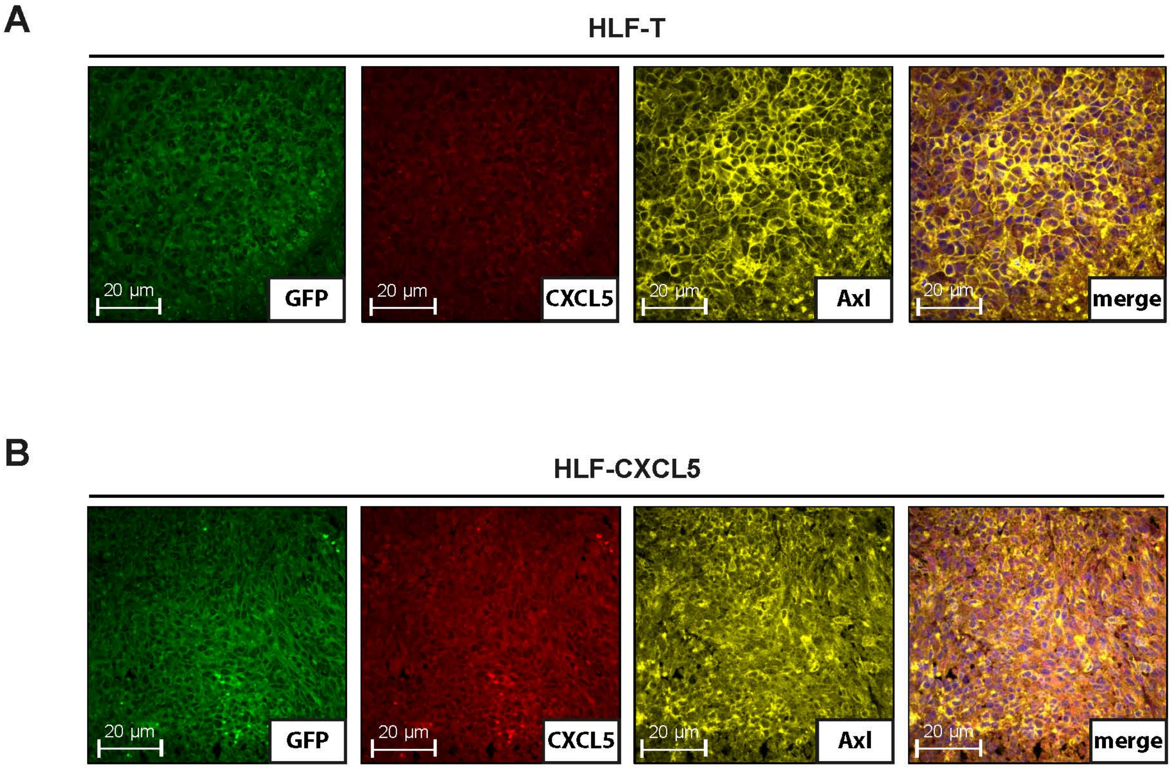

Haider et al, Supporting Figure S7

A

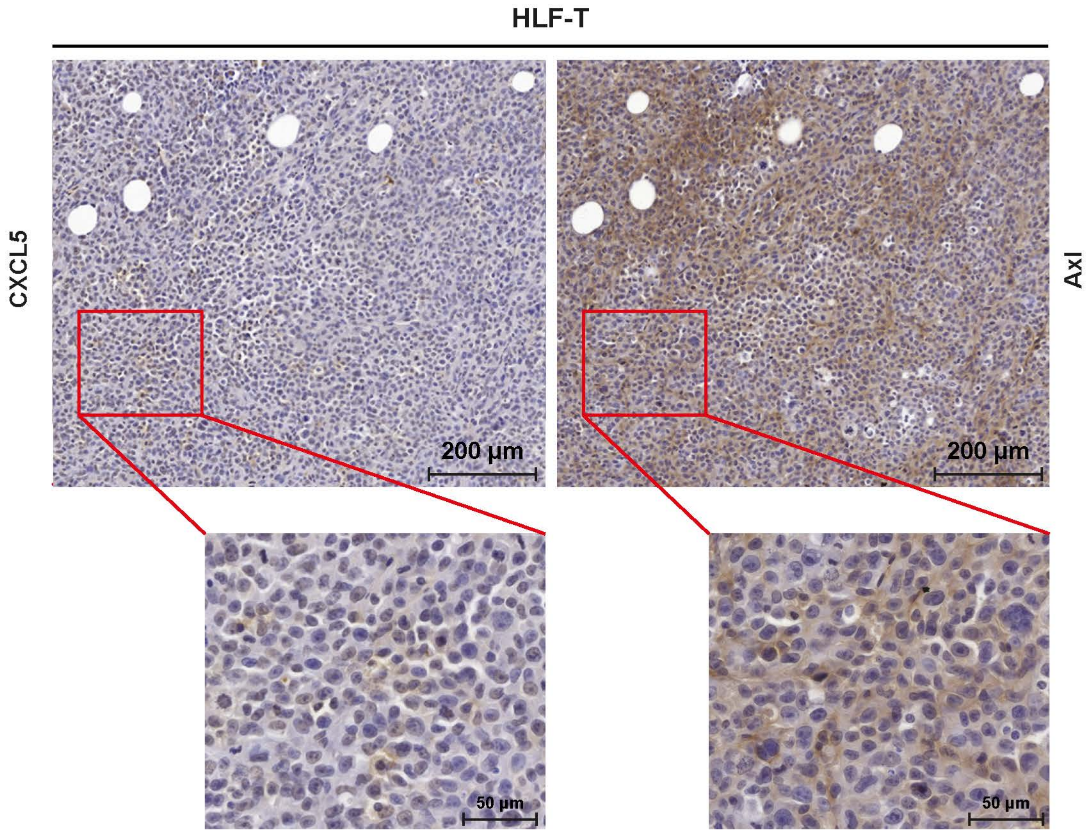

B

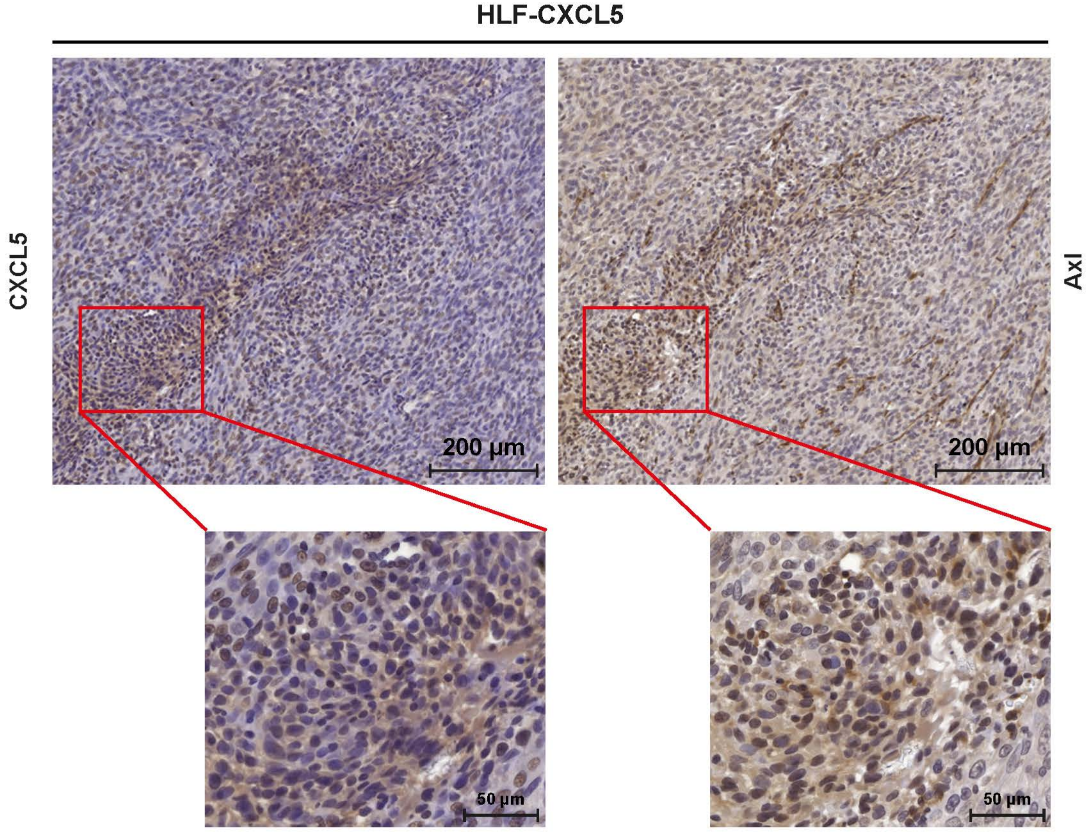

Haider et al, Supporting Figure S8

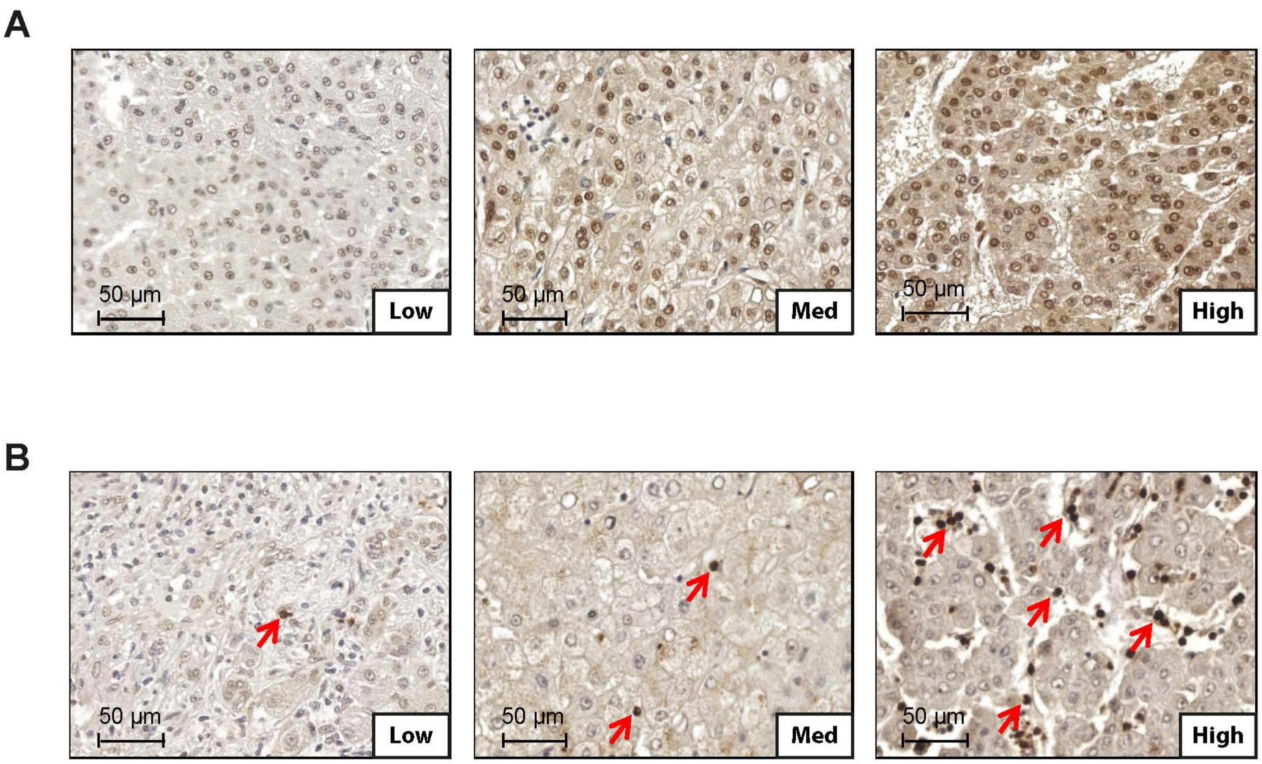

Haider et al, Supporting Figure S9

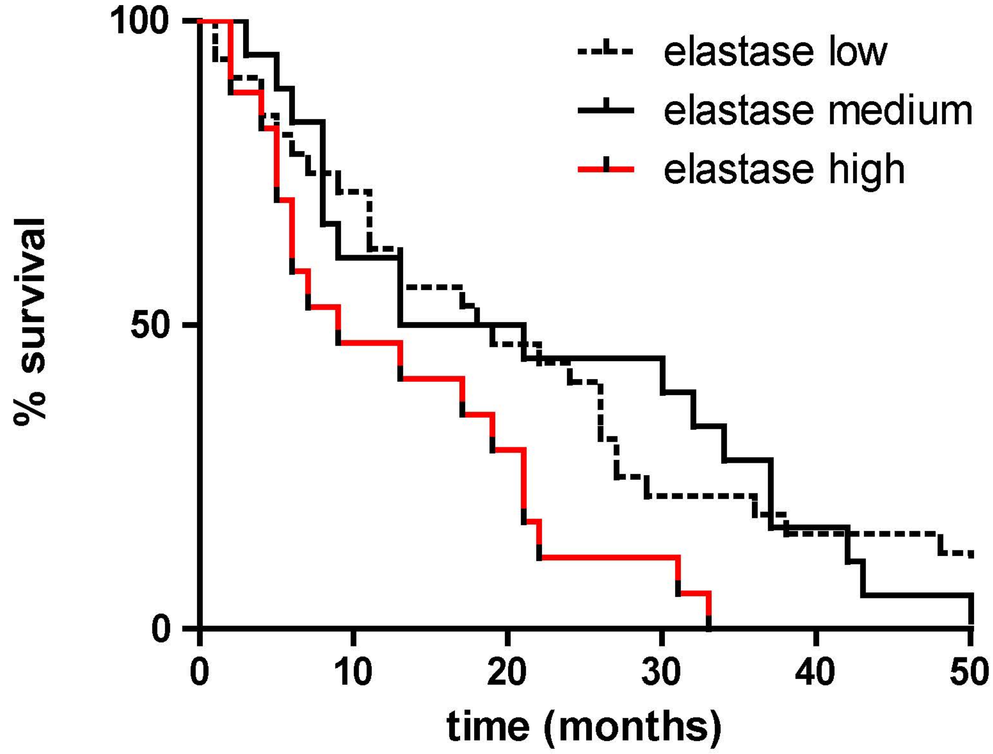

Haider et al, Supporting Table 1

qRT-PCR primer sequences

mRNA		Primer sequence	Product length (bp)
**TGF-β1**			
	F	5'-GCAGTACAGCAAGGTCCTG-3'	107
	R	5'-CGTAGTACACGATGGGCAG-3'	
**CXCL5**			
	F	5'-AGGTGGAAGTGGTAGCCTCC-3'	147
	R	5'-AAACTTTTCCATGCGTGCTC-3'	
**C15 orf48**			
	F	5'-GTGGTGTTCATGACTGTGGC-3'	112
	R	5'-CAGTTTCCCAAGGTTCTGGA-3'	
**CT83**			
	F	5'-ACTCCTAGCGAGCAGCATTC-3'	179
	R	5'-CCCGAGAGAGGTCGTAGACT-3'	
**DNER**			
	F	5'-TGGGGGTGTGTGCACCT-3'	129
	R	5'-CAAGGATCTGCAACAAGCTG -3'	
**MFAP**			
	F	5'-AAATTTTTCAGCTGTCCTCTCTGA-3'	87
	R	5'-GGCAGGAATAGCAGGAAGAGGTA-3'	
**SLC22A15**			
	F	5'-ATTTCAGCAGCAGCTTCACC-3'	115
	R	5'-AAGCGATCTGAAAGCTGACC-3'	
**TCF21**			
	F	5'-AGCTACATCGCCCACTTGAG-3'	119
	R	5'-TTCAGGTCACTCTCGGGTTT-3'	

F, forward. R, reverse

Haider et al, Supporting Table 2

Supporting Table 2. Correlation of CXCL5 expression with clinicopathological parameters

Variable	Number of cases	CXCL5		OR	CI 95%	*p*
		med/high	low			
**Age (years)**						
<56	47	26 (55.32%)	21 (44.68%)	0.696	0.318-1.527	0.427
≥56	54	25 (46.30%)	29 (53.7%)			
**Gender**						
Male	89	45 (50.56%)	44 (49.44%)	0.815	0.232-2.865	1.000
Female	11	5 (45.45%)	6 (54.55%)			
**HBV status**						
Negative	89	44 (49.44%)	45 (50.56%)	1.227	0.349-4.316	1.000
Positive	11	6 (54.55%)	5 (45.45%)			
**HCV status**						
Negative	67	35 (52.24%)	32 (47.76%)	0.762	0.330-1.758	0.671
Positive	33	15 (45.45%)	18 (54.55%)			
**Cirrhosis**						
No	11	4 (36.36%)	7 (63.64%)	1.872	0.511-6.848	0.525
Yes	89	46 (54.69%)	43 (48.31%)			
**Tumor stage >III**						
No	54	25 (46.30%)	29 (53.70%)	1.381	0.627-3.040	0.547
Yes	46	25 (54.35%)	21 (45.65%)			
**Vascular invasion**						
No	76	38 (50.00%)	38 (55.32%)	0.923	0.374-2.280	1.000
Yes	25	12 (48.00%)	13 (52.00%)			
**Lymph node metastasis**						
No	97	49 (50.52%)	48 (49.48%)	0.490	0.043- 5.582	1.000
Yes	3	1 (33.33%)	2 (66.67%)			

CI, confidence interval. OR, odds ratio. HBV, hepatitis B virus. HCV, hepatitis C virus.

***, $p < 0.001$; **, $p < 0.01$; *, $p < 0.05$

Haider et al, Supporting Table 3

Supporting Table 3. Correlation of elastase expression with clinicopathological parameters

Variable	Number of cases	Elastase med/high	Elastase low	OR	CI 95%	*p*
**Age (years)**						
<56	47	30 (63.83%)	17 (36.17%)	0.708	0.318-1.579	0.424
≥56	54	30 (55.56%)	24 (44.44%)			
**Gender**						
Male	89	52 (58.43%)	37 (41.57%)	1.245	0.340-4.564	1.000
Female	11	7 (63.64%)	4 (36.36%)			
**HBV status**						
Negative	89	56 (62.92%)	33 (37.08%)	0.221	0.055-0.892	0.047*
Positive	11	3 (27.27%)	8 (72.73%)			
**HCV status**						
Negative	67	38 (56.72%)	29 (43.28%)	1.336	0.566-3.151	0.526
Positive	33	21 (63.64%)	12 (36.36%)			
**Cirrhosis**						
No	11	7 (63.64%)	4 (36.36%)	0.803	0.219-2.943	1.000
Yes	89	52 (58.43%)	37 (41.57%)			
**Tumor stage >III**						
No	54	32 (59.26%)	22 (40.74%)	0.977	0.439-2.173	1.000
Yes	46	27 (58.70%)	19 (41.30%)			
**Vascular invasion**						
No	76	45 (59.21%)	31 (40.79%)	1.033	0.411-2.597	1.000
Yes	25	15 (60.00%)	10 (40.00%)			
**Lymph node metastasis**						
No	97	59 (60.82%)	38 (39.18%)	0.322	0.028- 3.676	0.562
Yes	3	1 (33.33%)	2 (66.67%)			

CI, confidence interval. OR, odds ratio. HBV, hepatitis B virus. HCV, hepatitis C virus.

***, $p < 0.001$; **, $p < 0.01$; *, $p < 0.05$

Haider et al, Supporting Table 4

Supporting Table 4. Univariate and multivariate overall survival and recurrence-free survival analysis

Variable	Overall survival			Recurrence-free					
	Number of cases	Average survival (mo)	*p*	Number of cases	No-recurrence	Recurrence	OR	CI 95 %	*p*
				univariate analysis					
**Expression levels**									
med-high CXCL5	31	667 ± 137	0.922	50	34	16	1.244	0.341-1.891	0.667
low CXCL5	25	680 ±137		51	37	14			
med-high elastase	32	667 ± 168	0.629	41	23	18	3.130	0.132-0.772	0.014*
low elastase	24	685 ± 86		60	48	12			
				multivariate analysis					
**Expression levels**									
low CXCL5/ med-high elastase	11	669 ± 185	0.986	26	3	23	9.061	0.026- 0.468	0.002**
low CXCL5/ low elastase	14	666 ± 96		24	13	11			
med CXCL5/ med-high elastase	11	670 ± 139	0.575	20	7	13	0.557	0.368- 8.748	0.700
med CXCL5/ low elastase	8	700 ± 94		13	3	10			
high CXCL5/ med-high elastase	2	663 ± 174	0.753	14	2	12	6.000	0.014- 1.963	0.197
high CXCL5/ low elastase	10	748 ± 15		4	2	2			

Mo, months. CI, confidence interval. OR, odds ratio. ***, $p < 0.001$; **, $p < 0.01$; *, $p < 0.05$
